# Lithium Carbonate Conversion to Lithium Hydroxide Using Calcium Hydroxide: Equilibrium is Governed by Vaterite Formation

**DOI:** 10.1021/acs.inorgchem.5c04057

**Published:** 2025-11-21

**Authors:** Péter Török, Ilona Halasiné-Varga, Laurent Duvivier, Maha Maimi, Olivier Hubert, Susanna Minnaar, Pál Sipos, Chris du Plessis, Bence Kutus

**Affiliations:** † Department of Molecular and Analytical Chemistry, 37442 University of Szeged, Dóm tér 7-8, H-6720 Szeged, Hungary; ‡ Lhoist, Business Innovation Centre, Rue de l’Industrie 31, B-1400 Nivelles, Belgium

## Abstract

The conversion of lithium carbonate (Li_2_CO_3_) with calcium hydroxide (Ca­(OH)_2_) is a cornerstone industrial process for synthesizing lithium hydroxide (LiOH), a critical precursor for high-performance cathodes in advanced lithium-ion batteries. Achieving a high Li_2_CO_3_-to-LiOH yield is crucial for efficient industrial processing. However, despite its industrial relevance, the influence of concentration and equilibrium conditions on this conversion reaction remains insufficiently explored. To address this gap, we investigated the conversion reaction by varying the Li_2_CO_3_ concentration and the Ca­(OH)_2_:Li_2_CO_3_ molar ratio. Near-complete conversion yields occur below the maximum concentration threshold of 1.6 mol L^–1^ LiOH, while yields diminish above this limit. Sequential reaction experiments confirm that the system adheres to Le Chatelier’s principle, and reverse reactions initiated from LiOH and CaCO_3_ demonstrate true equilibrium behavior. Furthermore, backward reactions involving distinct CaCO_3_ polymorphs reveal different equilibrium states. Notably, the presence of vaterite alongside calcite significantly affects the equilibrium concentration of LiOH, underscoring the role of solid-phase composition in governing reaction thermodynamics. These findings provide a deeper understanding of the causticization mechanism and offer actionable insights for optimizing LiOH production in industrial settings.

## Graphical abstract

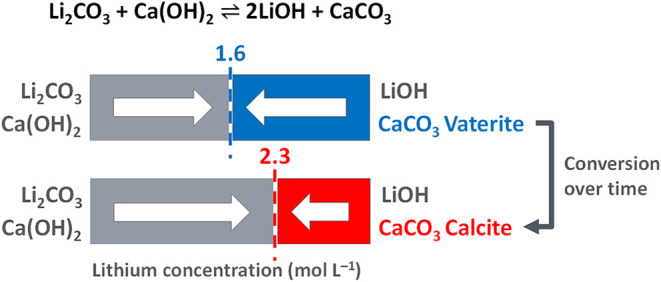

## Introduction

Lithium carbonate, Li_2_CO_3_, originating from processing lithium brines, hard rock lithium minerals (e.g., β-spodumene) or lithium-containing clays and micas,^1–6^ is a sparingly soluble salt,^1,6–10^ primarily used to produce cathodes (e.g., LiCoO_2_, LiFePO_4_)^11–14^ for Li-ion batteries. It is also used in various glass and cement formulations for the electrolytic production of aluminum, and in pharmaceutical applications.^1,8^ Li_2_CO_3_ is the most important precursor for the production of lithium hydroxide monohydrate, LiOH·H_2_O,^1–3,5,15,16^ a critical component of lithium nickel cobalt oxide (LiNi_*x*_Co_1–*x*_)^17^ and lithium nickel manganese cobalt oxide (Li-NMC, LiNi_*x*_Mn_*y*_Co_1–*x*–*y*_O_2_)^16–20^ cathodes used in electric vehicle batteries. The application of LiOH allows for employing lower synthesis temperatures, a higher degree of crystallinity, greater structural purity, and higher energy density in NMCs.^20,21^ In addition, the reaction between LiOH and HF yields LiF,^22^ a precursor of LiPF_6_, which is the primary electrolyte of rechargeable Li batteries.^1^ Other industrial applications of LiOH include the production of lubricating greases, cements, and CO_2_ adsorbents.^1^

At industrial scale, LiOH is produced mainly via the conversion of Li_2_CO_3_, using calcium hydroxide, Ca­(OH)_2_.^1,3,5,15,16,23–26^

1 $${\mathrm{Li}}_{2}{\mathrm{CO}}_{3\left(s\right)}+\mathrm{Ca}{\left(\mathrm{OH}\right)}_{2\left(s\right)}\to 2{\mathrm{LiOH}}_{\left(\mathrm{aq}\right)}+{\mathrm{CaCO}}_{3\left(s\right)}$$

The soluble LiOH product of this metathesis reaction, after solid–liquid separation and purification, is subjected to evaporation and crystallization to yield LiOH·H_2_O. Other methods for producing LiOH include the direct reaction between β-spodumene and CaCO_3_ ^1,2^ as well as membrane electrolysis,^23^ or direct chemical/electrochemical conversion of Li_2_SO_4_ and LiCl.^27–29^

The metathesis or causticization reaction in eq 1 is the main commercially deployed method for the preparation of LiOH,^1,3^ due to the relatively low cost and availability of Ca­(OH)_2_, and the mild conditions required for the reaction to proceed.

This causticization process is challenged by the fact that it is characterized by a maximum LiOH concentration of 3.5 wt % or 1.5 mol L^–1^, above which some of the Li_2_CO_3_ and Ca­(OH)_2_ remain unreacted.^1,3,15,23,24,28^ Typically, the maximum conversion yield of Li_2_CO_3_ is 95% even with extensive reaction times (>2 h).^1^ Such conversion has been reported for a dilute slurry containing 1.9 g L^–1^ Li^+^ (as suspended Li_2_CO_3_),^24^ whereas for 15 g L^–1^ Li^+^, a low conversion yield of 53% has been found at 25 °C, which increased only to 60% at 100 °C.^25^ Our findings from scoping experiments are in line with these results, indicating that the conversion depends heavily on the initial Li^+^ concentration in suspension. The reason for the dramatic decrease in LiOH yield is still unclear from current literature, although solubility suppression of both Li_2_CO_3_ and Ca­(OH)_2_ by the forming LiOH solution seems plausible.^1,3,24,28^ In addition, adsorption of Ca^2+^ ions onto Li_2_CO_3_ crystals and an intermediate Ca_*x*_Li_2–2*x*_CO_3_ mixed phase at 25 °C has also been suggested, contrary to the formation of CaCO_3_ via simple dissolution–precipitation mechanism observed at 100 °C.^25^

The increased global demand for LiOH necessitates a fundamental understanding of the causticization reaction in eq 1. To this end, we studied this process by varying the initial concentration of Li_2_CO_3_ and the Ca­(OH)_2_:Li_2_CO_3_ molar ratio at 30 °C. We find that the plateau value of LiOH molar concentration after 2 h is 36 g L^–1^ or 1.5 mol L^–1^ LiOH. Starting from CaCO_3_ suspended in concentrated LiOH solutions, the backward reaction also takes place, confirming that the causticization is a true equilibrium process, and no intermediate mixed carbonate phases need to be invoked to explain the observed yields. Strikingly, the backward reaction results in two very different LiOH concentrations providing sound evidence for the existence of two equilibrium states, which in turn are governed by the formation of vaterite.

## Results and Discussion

### Time-Dependence of the Forward Process

First, we studied the time-dependence of the causticization reaction via conductometry, applying a Ca­(OH)_2_:Li_2_CO_3_ molar ratio, φ (as %), of 100%, initial total Li^+^ concentration, *c*_Li^+^,0_, of 10 g L^–1^ (equivalent to 53.23 g Li_2_CO_3_ added to 1 L water), and total contact times ranging from 1 to 4 h (*T* = 30 °C). Figure 1a shows that following the addition of Ca­(OH)_2_, the conductivity, κ, of equilibrated Li_2_CO_3_ suspensions (17.5 mS cm^–1^) increases steeply and rapidly, indicating the exchange of CO_3_^2–^ to OH^–^ ions according to the metathesis reaction in eq 1. This marked increase in κ is due to the fact that LiOH has ca. 2.2 times greater limiting equivalent molar conductivity than Li_2_CO_3_ ^6,30–32^ and has much higher solubility than the carbonate salt in water,^1–6,33,34^ e.g., 29.6 vs 0.7 g/100 mL.^1,6^ Minor differences are seen between the different curves up to 100 s, shown in the inset of Figure 1a. Initial short-term scatter immediately after the addition of Ca­(OH)_2_ is an expected consequence of mixing a solid reactant into solution. Over longer time-scales, however, all curves are virtually identical, indicating the overall good reproducibility of the reactions. (The repeatability is also demonstrated by replicate measurements; see Figures S1–S3 and discussion in Supporting Note 1 in the SI.) After 30 min, κ approaches a plateau of ∼180 mS cm^–1^ after 4 h, indicating the slowdown of the reaction. Contact times of 2 h are sufficient for the reaction to reach >98% of this maximum, also demonstrated by the dashed line in the inset of Figure 1a. This is also reflected by the observation that the measured total concentration OH^–^ ions, [OH^–^]_T_, increases by 6% upon increasing the reaction time from 1 to 2 h, but further increase is marginal (0.8%); see Table S1 in Supporting Note 2. Therefore, 2 h reaction time was chosen for most experiments, which is also relevant for large-scale industrial processing.

**Figure 1:**
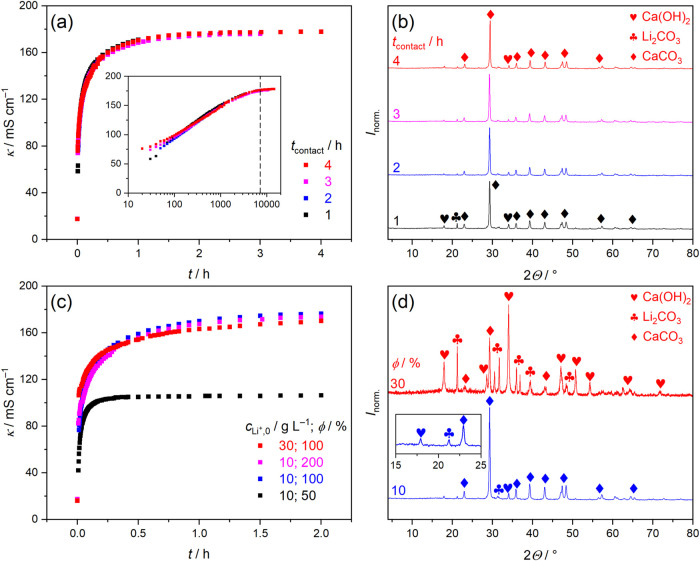
(a) Variation of conductivity, κ, as a function of reaction time of Li_2_CO_3_ suspensions (*c*_Li^+^,0_ = 10 g L^–1^) upon addition of equimolar Ca­(OH)_2_, at different contact times and (*T* = 30 °C). The inset shows the same plots on logarithmic scale; the vertical dashed line corresponds to 7200 s. (b) Powder X-ray diffractograms of solids corresponding to reactions of panel (a). (c) Variation of κ for different initial Li^+^ concentrations, *c*_Li^+^,0_, of 10 and 30 g L^–1^, and different Ca­(OH)_2_:Li_2_CO_3_ molar ratios, φ, of 50–200%. The contact time was 2 h in each case. (d) Diffractograms of solids corresponding to reactions of panel (c). Symbols represent Ca­(OH)_2_ (PDF #84–1264), Li_2_CO_3_ (PDF #83–1454) and product CaCO_3_ (calcite polymorph, PDF #83–1762).^35^ The XRD data were normalized such that the maximum intensity within each data set is 1.

X-ray powder diffractometry analyses of the solid products of the above reactions are shown in Figure 1b. Using literature diffractograms for Li_2_CO_3_, Ca­(OH)_2_, and the calcite form of CaCO_3_,^35^ all diffraction peaks of these solid phases can be identified. There is no evidence that a new crystalline intermediate phase forms after 2 h. Nevertheless, partial incorporation of Li^+^ ions into the structure of Ca­(OH)_2_ ^36^ or that of Ca^2+^ ions into Li_2_CO_3_ ^25^ cannot be completely excluded, albeit they are expected to give rise to minor shifts or variations of the diffraction peaks’ intensities. Overall, the calcite polymorph of CaCO_3_ is detected as the dominant phase and the reactants are gradually consumed on increasing the contact time in line with the leveling off of κ. Further diffractograms of replicate measurements are shown in Figure S4 in Supporting Note 1.

The conductometric curves with suspensions at different φ (50–200%) at *c*_Li^+^,0_ = 10 g L^–1^ are compared in Figure 1c. The increase in κ at φ = 50% is steeper before the plateau as compared to 100%, signaling that the former reaction proceeds relatively faster. Further, the maximum conductivity after 2 h, κ_max_ at φ = 50% is expected to be half of κ_max_ at φ ≥ 100%, since the total molar concentration of forming LiOH, [LiOH]_T_, at φ = 50% (excess Li_2_CO_3_) should be half of [LiOH]_T_ at φ ≥ 100% (equimolar and excess Ca­(OH)_2_). This is clearly not the case, as κ_max_ = 107 mS cm^–1^ (φ = 50%), whereas κ_max_ = 176 (φ = 100%) and 174 mS cm^–1^ (φ = 200%), which can be elucidated by two phenomena. First, Kohlrausch’s law states that the molar conductivity, Λ, decreases linearly with the square root of [LiOH]_T_. Since κ_max_ = Λ_LiOH_·[LiOH]_T_, κ_max_ at φ ≥ 100% will be smaller than 2κ_max_ at φ = 50%. Second, in case of heterogeneous systems, higher volume concentration of suspended insulators hampers ion migration,^37^ again decreasing κ in the order of φ = 50% > 100% > 200%.

Last, increasing the reactant concentrations from *c*_Li^+^,0_ = 10 to 30 g L^–1^, κ_max_ remains virtually unchanged (176 vs 170 mS cm^–1^); see Figure 1c. In addition to the decrease induced by higher volume concentration of solid particles,^37^ this similarity indicates much lower conversion for *c*_Li^+^,0_ = 30 g L^–1^than for 10 g L^–1^. The diffractograms in Figure 1d indeed show that both Li_2_CO_3_ and Ca­(OH)_2_ are barely visible at *c*_Li^+^,0_ = 10 g L^–1^, whereas they remain largely unreacted at *c*_Li^+^,0_ = 30 g L^–1^.

### Concentration-Dependence of the LiOH Yield

Next, the dependence of the conversion yield, *X*, on φ, based on [OH^–^]_T_ or [LiOH]_T_, was studied at 30 °C. *X* was calculated as the ratio of [OH^–^]_T_ to [OH^–^]_T,max_

2 $$X=\frac{{\left[\mathrm{LiOH}\right]}_{T}}{{\left[\mathrm{LiOH}\right]}_{T,\max}}\cdot 100=\frac{{\left[{\mathrm{OH}}^{-}\right]}_{T}}{{\left[{\mathrm{OH}}^{-}\right]}_{T,\max}}\cdot 100$$

where [OH^–^]_T,max_ is the maximum concentration of OH^–^ ions forming during the reaction if *X* = 100%. Note that the calculation of *X* is complicated by the fact that the density of the solution changes during the reaction. (The way of calculating *X* is discussed in detail in Supporting Note 3.) Furthermore, [LiOH]_T_ = [OH^–^]_T_ if the concentration of Ca^2+^ ions is negligible compared to that of Li^+^ ions. This has been confirmed by ICP-MS measurements for time-dependent experiments (*c*_Li^+^,0_ = 10 g L^–1^); see Table S1 and discussion in Supporting Note 2.

Figure 2a shows *X* as a function of φ for *c*_Li^+^,0_ = 5 and 10 g L^–1^, respectively. Accordingly, *X* has a minimum at φ = 100%, indicating higher conversion after 2 h if either of the two reactants is in excess. The higher conversion at φ = 50% as compared to 100% is consistent with the faster relative reaction rate deduced from conductometry (Figure 1c). Typically, excess concentration of M or X of an MX salt decreases its solubility in thermodynamic equilibrium, known as the common-ion effect. This effect has also been reported to decrease the rate of salt dissolution.^38,39^ Hence, it is reasonable to assume that rising concentrations of Li^+^ and OH^–^ ions in solution up to φ = 100% will increasingly slow down the dissolution of both Li_2_CO_3_ and Ca­(OH)_2_, thereby resulting in lower yields. Further, *X* is lower for *c*_Li^+^,0_ = 10 g L^–1^ than for 5 g L^–1^ at all compositions. Since [LiOH]_T,max_ for 10 g L^–1^ is double the value for *c*_Li^+^,0_ = 5 g L^–1^, it gives rise to a more pronounced common-ion effect thus lower *X*.

**Figure 2:**
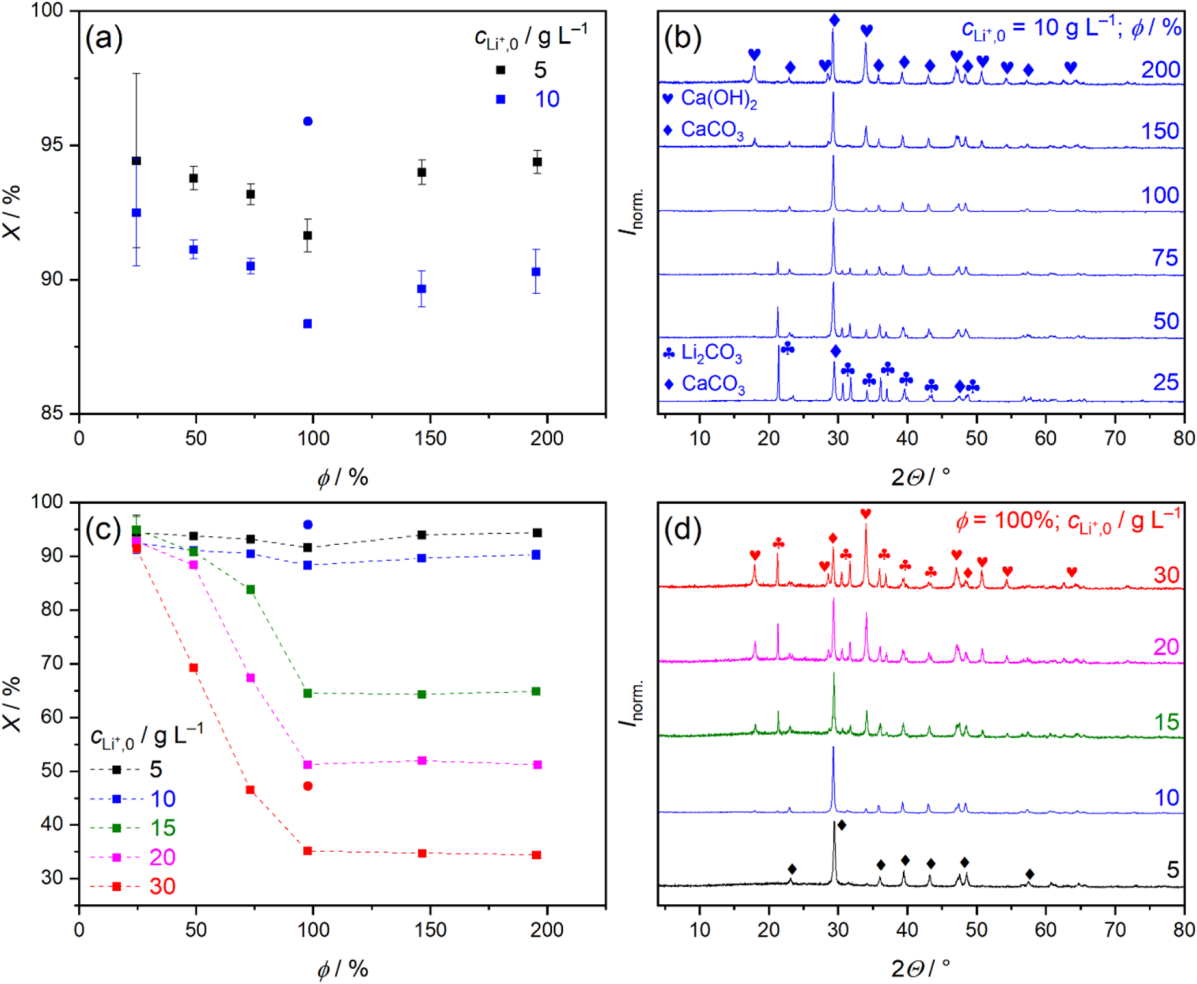
(a) Variation of conversion, *X*, as a function of Li_2_CO_3_:Ca­(OH)_2_ molar ratio, φ, at different initial Li^+^ concentrations, *c*_Li^+^,0_, of 5 and 10 g L^–1^ (*T* = 30 °C). Contact times are 2 h (squares) or 2 weeks (blue circle). All data were obtained from triplicate measurements; the error bars represent the standard deviation of the averages. (b) Powder X-ray diffractograms of solids corresponding to reactions of panel (a); *c*_Li^+^,0_ = 10 g L^–1^. (c) Variation of *X* as a function of φ at *c*_Li^+^,0_ = 5–30 g L^–1^. The dashed lines serve as a guide to the eye. (d) Diffractograms of solids corresponding to reactions of panel (c); φ = 100%. Symbols represent Ca­(OH)_2_ (PDF #84–1264), Li_2_CO_3_ (PDF #83–1454) and product CaCO_3_ (calcite polymorph, PDF #83–1762).^35^ The XRD data were normalized such that the maximum intensity within each data set is 1.

Above φ = 100%, [LiOH] cannot increase further significantly due to the stoichiometric constraint in eq 1. Thus, the slightly higher conversions are probably due to the larger total surface of Ca­(OH)_2_, being a critical parameter for such heterogeneous reaction. Nevertheless, the values of *X* are equal or higher than 88% for both series already after 2 h, indicating close-to-equilibrium states. Such high conversions translate to virtual disappearance of either Ca­(OH)_2_ in excess Li_2_CO_3_ or that of Li_2_CO_3_ in excess Ca­(OH)_2_, or the absence of both at equimolar composition, demonstrated by the X-ray diffractograms in Figure 2b. After 2 weeks, *X* for *c*_Li^+^,0_ = 10 g L^–1^ increases from 88 to 96%, matching the industrial maximum conversion (95%).^1^

The values of *X* for *c*_Li^+^,0_ = 5, 10, 15, 20, and 30 g L^–1^ are shown in Figure 2c, whereas data for *X*, [OH^–^]_T_, [CO_3_^2–^]_T_, and density are tabulated in Table S2 in Supporting Note 3. Strikingly, the latter three, more concentrated suspensions exhibit a dramatic decrease in *X*, which reaches minima of 64% (15 g L^–1^), 50% (20 g L^–1^) and 35% (30 g L^–1^) after 2 h. In line with this finding, *X* = 50% has been reported for *c*_Li^+^,0_ = 15 g L^–1^ after 1 h.^25^ Furthermore, XRD traces in Figure 2d show that at φ = 100%, the residual amount of reactants increases in the order of 5 < 10 < 15 < 20 ≈ 30 g L^–1^ Li^+^, with no diffraction peaks of Li_2_CO_3_ or Ca­(OH)_2_ for 5 g L^–1^.

Figure 3a shows a linear increase in [OH^–^]_T_ at *c*_Li^+^,0_ = 5 and 10 g L^–1^ up to φ = 100%, and at 15 and 20 g L^–1^ up to φ = 50%, indicating near-quantitative conversion, which can be quantified in terms of enhancement factors; see Table S3 and discussion in Supporting Note 4. However, [OH^–^]_T_ reaches a maximum value of 1.48 mol L^–1^ above these compositions, mirroring the parallel decrease in *X* (Figure 2a). That is, at least for 2 h, ∼1.5 mol L^–1^ is the approximate maximum concentration attainable, i.e., 3.4 wt % LiOH (based on the measured density; Table S2), in line with the literature-reported value of 3.5 wt %.^1,3,15,23,24,28^

**Figure 3:**
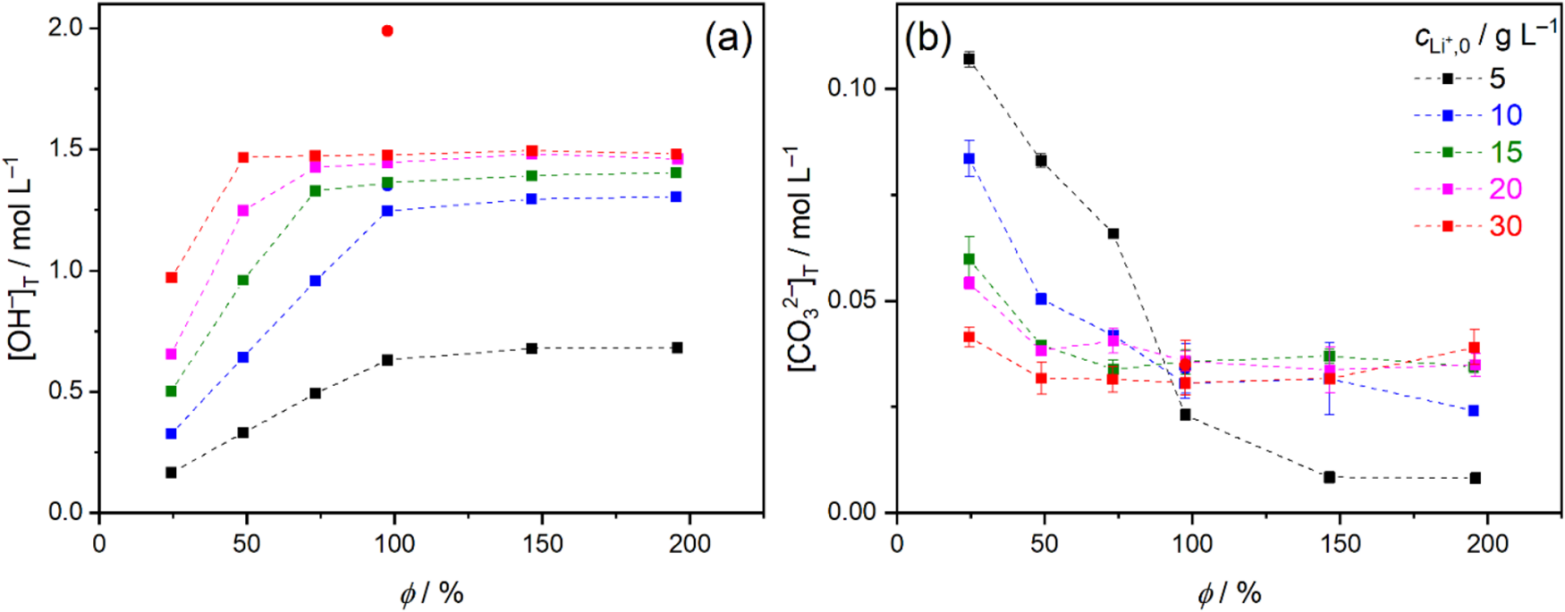
Variation of the total concentration of (a) OH^–^, [OH^–^]_T_, and (b) that of CO_3_^2–^ ions, [CO_3_^2–^]_T,0_, as a function of Li_2_CO_3_:Ca­(OH)_2_ molar ratio, φ, at different initial Li^+^ concentrations, *c*_Li^+^,0_ = 5–30 g L^–1^ (*T* = 30 °C). Reaction times were 2 h (squares) or 2 weeks (circles). All data were obtained from triplicate measurements; the error bars represent the standard deviation of the averages. In panel (a), error bars are not larger than the size of the symbols. The dashed lines serve as a guide to the eye.

Applying 2 weeks of contact time, [LiOH]_T_ can be increased from 1.25 to 1.35 mol L^–1^ for *c*_Li^+^,0_ = 10 g L^–1^, which clearly reflects the effect of longer equilibration time. Conversely, the 35% increase of 1.48 to 2.0 mol L^–1^ for *c*_Li^+^,0_ = 30 g L^–1^ cannot be explained solely by longer reaction time and will be discussed later.

Finally, parallel to the increase in [OH^–^]_T_, a steep decrease in [CO_3_^2–^]_T_ as a function of φ is observed in Figure 3b. The decrease stems predominantly from the common-ion effect exerted by excess Li^+^ ions on the solubility of Li_2_CO_3_: as [Li^+^]_T_ increases, [CO_3_^2–^] decreases quadratically, which can be expressed via the solubility product of Li_2_CO_3_, *K*_sp_(Li_2_CO_3_)­

3 $${\left[{{\mathrm{CO}}_{3}}^{2-}\right]}_{T}=\frac{K_{\mathrm{sp}}\left({\mathrm{Li}}_{2}{\mathrm{CO}}_{3}\right)}{{{\left[{\mathrm{Li}}^{+}\right]}_{T}}^{2}}$$

For simplicity, an ideal solution with zero ionic strength is assumed, thus thermodynamic activities can be replaced by molar concentrations, according to the Debye–Hückel theory^40^. This effect also explains that up to φ = 100%, [CO_3_^2–^]_T_ decreases with increasing *c*_Li^+^,0_. The same trend is also expected for [Ca^2+^]_T_, due to the common-ion effect from OH^–^ ions.

However, the order appears to be reversed in Ca­(OH)_2_ excess. The most plausible reason behind this phenomenon is the possible ion-pairing between Li^+^ and CO_3_^2–^ ions

4 $${{\mathrm{Li}}^{+}}_{\left(\mathrm{aq}\right)}+{{{\mathrm{CO}}_{3}}^{2-}}_{\left(\mathrm{aq}\right)}⇌{{{\mathrm{LiCO}}_{3}}^{-}}_{\left(\mathrm{aq}\right)}$$

Upon increasing [LiOH]_T_ or [Li^+^]_T_, the degree of ion association, and thus the solubility of Li_2_CO_3_, will increase, overcoming the decrease arising from the common-ion effect. This is most obvious at φ = 200%, where despite rising [Li^+^]_T_, [CO_3_^2–^]_T_ also increases as a function of *c*_Li^+^,0_, indicating ion pairing. ([CO_3_^2–^]_T_ data are listed in Table S2).

Ion pairing is primarily governed by electrostatic attractions. Since the LiOH^0^ ion-pair has already been reported,^31,41^ even stronger association for doubly charged CO_3_^2–^ ions are expected. Surprisingly, the literature appears to lack information on the ion-pairing equilibria of Li_2_CO_3_ solutions. Quantitation of such ion association should be possible indirectly by studying the protonation steps of CO_3_^2–^ in the presence of different background electrolytes, or directly via dielectric spectroscopy, sensitive to the formation and hydration state of dipolar ion-pairs. Both methods have been applied to probe the degree of association of the NaCO_3_^–^ species.^42,43^ As for the ion-pairing constant, *K* ^IP^, it can be speculated that *K* ^IP^(LiCO_3_^–^) > *K* ^IP^(NaCO_3_^–^) = 9.1–14.1,^42,43^ based on the smaller ionic radius and higher charge density of Li^+^ as compared to Na^+^.

Another aspect of the causticization worth considering is that the solubility of CaCO_3_ is very small but not infinitely small,^44^ and will be further enhanced by ion pairing. Nevertheless, even in the absence of ion pairing, [CO_3_^2–^]_T_ will not be zero, hence *X* = 100% cannot be attained, as all CO_3_^2–^ ions in solution count as unreacted Li_2_CO_3_. The conversion of 96% obtained at *c*_Li^+^,0_ = 10 g L^–1^ after 2 weeks can be regarded as the maximum achievable yield.

### Characterization of the Equilibrium

The above data for *X* and [OH^–^]_T_ corresponding to *c*_Li^+^,0_ = 15–30 g L^–1^ show incomplete transformation of Li_2_CO_3_ to LiOH even after long reaction times, suggesting that the causticization reaction is an equilibrium process

5 $${\mathrm{Li}}_{2}{\mathrm{CO}}_{3\left(s\right)}+\mathrm{Ca}{\left(\mathrm{OH}\right)}_{2\left(s\right)}⇌2{\mathrm{Li}}_{\left(\mathrm{aq}\right)}^{+}+{\mathrm{OH}}_{\left(\mathrm{aq}\right)}^{-}+{\mathrm{CaCO}}_{3\left(s\right)}$$

where LiOH is considered as a strong electrolyte, dissociating completely to Li^+^ and OH^–^ ions. Such equilibrium arises naturally from the fact that the overall reaction can be composed of the three solubility equilibria of Li_2_CO_3_, Ca­(OH)_2_ and CaCO_3_. As such, the equilibrium constant, *K*_1_, reads as

6 $$K_{1}=\frac{K_{\mathrm{sp}}\left({\mathrm{Li}}_{2}{\mathrm{CO}}_{3}\right)\cdot K_{\mathrm{sp}}\left(\mathrm{Ca}{\left(\mathrm{OH}\right)}_{2}\right)}{K_{\mathrm{sp}}\left(\mathrm{Ca}{\mathrm{CO}}_{3}\right)}={{(a}_{{\mathrm{Li}}^{+}}a_{{\mathrm{OH}}^{-}})}^{2}$$

where *K*_sp_ is the solubility product of a given phase and *a*_X_ represents the thermodynamic activity of species *X*. The derivation of eq 6 is given in detail in Supporting Note 5.

Regarding the concentrations of cations and anions, the charge balance can be written as

7 $${\left[{\mathrm{Li}}^{+}\right]}_{T}+2{\left[{\mathrm{Ca}}^{2+}\right]}_{T}={\left[{\mathrm{OH}}^{-}\right]}_{T}+2{\left[{{\mathrm{CO}}_{3}}^{2-}\right]}_{T}$$

Due to the much lower solubility of both Ca­(OH)_2_ and CaCO_3_ as compared to Li_2_CO_3_ and especially to LiOH, [Ca^2+^]_T_ ≪ [Li^+^]_T_, as confirmed by ICP-MS measurements (Table S1). Further, [CO_3_^2–^]_T_/[OH^–^]_T_ ratios are less than 3% for each solution with [LiOH]_T_ ≈ 1.48 mol L^–1^ (Table S2). Thus, eq 7 reduces to [Li^+^]_T_ ≈ [OH^–^]_T_, and *K*_1_ can be approximated by the following formula

8 $$K_{1}\approx {\left(\gamma_{\pm ,\mathrm{LiOH}}\frac{{\left[\mathrm{LiOH}\right]}_{\mathrm{eq}}}{c^{ø}}\right)}^{4}\overset{\mathrm{def}}{=}{\left(a_{\pm ,\mathrm{LiOH}}\right)}^{4}$$

where γ_±,LiOH_ and *a*_±,LiOH_ are the mean activity coefficient and activity of LiOH; [LiOH]_eq_ is the molar concentration of LiOH in equilibrium, and *c* ^ø^ is 1 mol L^–1^ in each case. The method to obtain eq 8 is described in detail in Supporting Note 6.

Further, eqs 5 and 6 do not define which polymorph of CaCO_3_, calcite, aragonite, or vaterite,^44^ should be considered. Calcite is the most stable of the polymorphs with the lowest *K*_sp_,^44^ and its dominance is supported by the XRD traces (Figure 2b,d) as well. Nevertheless, further experiments showed that vaterite is also an important equilibrium solid, as discussed later.

The nearly quantitative conversion of Ca­(OH)_2_ in Li_2_CO_3_ excess or vice versa at *c*_Li^+^,0_ = 5 and 10 g L^–1^ is driven by the favorable precipitation of CaCO_3_, supported by the ∼90% yields see Figure 2a and Table S2. In these cases, only two solids (Li_2_CO_3_/CaCO_3_ or Ca­(OH)_2_/CaCO_3_) are present in equilibrium, and the high conversions can be characterized by equilibrium constants *K*_2_ and *K*_3_, which can be derived similarly to *K*_1_. (For more discussion, see Supporting Note 7).

In both cases, when (*a*_Li^+^_ *a*_OH^–^_)^2^ reaches a critical value, all three solids precipitate as equilibrium phases. (Thus, in a sense, *K*_1_ is itself a solubility product.) Under nonequilibrium conditions, as [Li^+^]_T_ and [OH^–^]_T_ rise, they lower the dissolution rate of Li_2_CO_3_ and Ca­(OH)_2_ markedly. (Such common-ion effect is absent for CaCO_3_, as there is no strong electrolyte which would introduce either Ca^2+^ or CO_3_^2–^ at large concentrations, e.g., CaCl_2_ or Na_2_CO_3_). As the reaction proceeds, the dissolution rate of Ca^2+^ ions from Ca­(OH)_2_ and that of CO_3_^2–^ ions from Li_2_CO_3_ will be equal to the rate of CaCO_3_ dissolution. This is when the system reaches dynamic equilibrium and can be described by *K*_1_ and [LiOH]_T_ ≈ 1.5 mol L^–1^.

### Proving the Equilibrium State by Sequential Reactions

Proving that the causticization reaction is a true equilibrium process requires further evidence. In this respect, *K*_1_ is related to the standard free energy of reaction, Δ_*r*_ *G*_1_^ø^

9 $$\Delta_{r}{G_{1}}^{⌀}=-\mathrm{RT}\ln K_{1}$$

Δ_*r*_ *G*_1_^ø^ is a state function, i.e., the equilibrium state is independent of the path the system has taken to reach it. To prove this, we carried out the original one-step reaction (*c*_Li^+^,0_ = 30 g L^–1^ and φ = 100%) in three steps in the following sequence: (1) addition of equimolar Ca­(OH)_2_ to an equilibrated Li_2_CO_3_ suspension with *c*_Li^+^,0_ = 10 g L^–1^, (2) addition of the remaining Li_2_CO_3_ to have *c*_Li^+^,0_ = 30 g L^–1^, and (3) addition of the remaining Ca­(OH)_2_ to again satisfy φ = 100%.

Figure 4a shows the rapid increase in κ upon adding equimolar Ca­(OH)_2_ to Li_2_CO_3_ (*c*_Li^+^,0_ = 10 g L^–1^, red symbols), due to the appearance of conducting Li^+^ and OH^–^ ions. After 2 h, κ closely matches that of a reference one-step reaction at *c*_Li^+^,0_ = 30 g L^–1^ and φ = 100% (black symbols; also shown in Figure 1c), indicating similar [LiOH]_T_ values (1.25 mol L^–1^ for *c*_Li^+^,0_ = 10 g L^–1^ vs 1.48 mol L^–1^ *c*_Li^+^,0_ = 30 g L^–1^).

**Figure 4:**
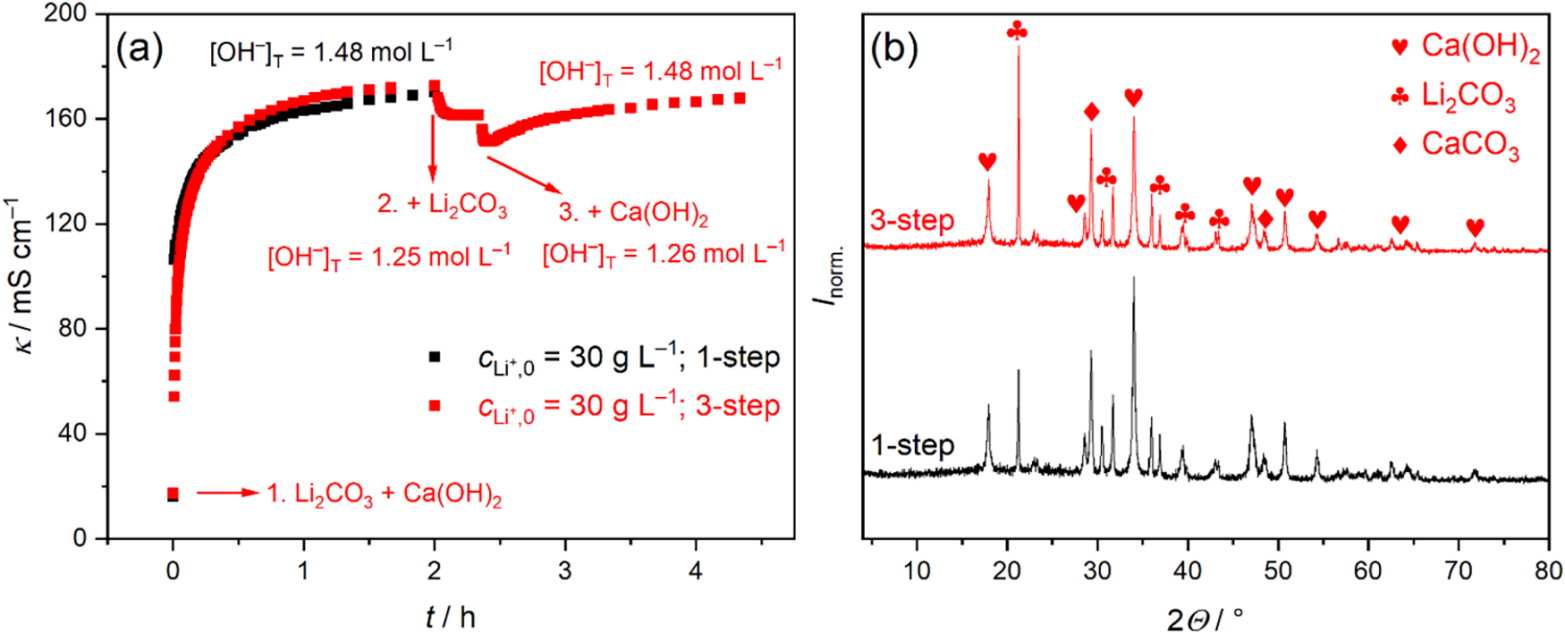
(a) Variation of conductivity, κ, as a function of reaction time of Li_2_CO_3_ suspensions upon addition of Ca­(OH)_2_ (*T* = 30 °C). Black symbols correspond to a reaction where Ca­(OH)_2_ was added to a Li_2_CO_3_ suspension with *c*_Li^+^,0_ = 30 g L^–1^ in one step. Red symbols represent the case where (1) equimolar Ca­(OH)_2_ was added to a Li_2_CO_3_ suspension with *c*_Li^+^,0_ = 10 g L^–1^, then (2) Li_2_CO_3_ with *c*_Li^+^,0_ = 20 g L^–1^ was added, and (3) equimolar Ca­(OH)_2_ was added. Therefore, the final lithium concentration was 30 g L^–1^ in both cases (with equimolar Ca­(OH)_2_). Also shown are the total concentrations of hydroxide, [OH^–^]_T_, at each step. (b) Powder X-ray diffractograms of solids corresponding to reactions of panel (a). Symbols represent Ca­(OH)_2_ (PDF #84–1264), Li_2_CO_3_ (PDF #83–1454) and product CaCO_3_ (calcite polymorph, PDF #83–1762).^35^ The XRD data were normalized such that the maximum intensity within each data set is 1.

In the second step, Li_2_CO_3_ is added to reach *c*_Li^+^,0_ = 30 g L^–1^, reducing φ to 33%, which causes κ to drop and then level off, indicating unchanged [LiOH]_T_. Indeed, [OH^–^]_T_ = 1.26 mol L^–1^ postaddition (based on an independent experiment), nearly identical to the 1.25 mol L^–1^ from step one. The drop in κ results again from introducing solid Li_2_CO_3_ acting as an insulator.^37^ (This is confirmed by repeating the first two steps without Ca­(OH)_2_; see Figure S5a in Supporting Note 8.) The reason for unaltered [OH^–^]_T_ is that most Ca­(OH)_2_ has already reacted at this point (*X* = 88%), and further LiOH formation would require extended reaction times (e.g., 2 weeks, Figure 2a).

In the third step, with *c*_Li^+^,0_ = 30 g L^–1^ and φ = 100%, adding the remaining Ca­(OH)_2_ again gives rise to an initial drop in κ, as also shown by experiments performed only with adding Ca­(OH)_2_ (see Figure S5b in Supporting Note 8). Unlike the previous step, both Li_2_CO_3_ and Ca­(OH)_2_ are now in excess. [LiOH]_T,max_ is 4.21 mol L^–1^, so *X* = 1.26/4.21·100 = 30%, slightly below the 35% from the one-step reaction (Figure 2c). The system thus shifts to restore equilibrium, evidenced by the increase in κ, consistent with Le Chatelier’s principle.

Expectedly, [LiOH]_T_ does not reach [LiOH]_T,max_ but plateaus at 1.48 mol L^–1^, exactly at the same value as for the one-step reaction. This match is also reflected by the similarity between the plateau values of κ (168 mS cm^–1^ for the three-step vs 170 mS cm^–1^ for the one-step process). In addition, repeating the above sequence of (i) Li_2_CO_3_ + Ca­(OH)_2_ + Li_2_CO_3_ + Ca­(OH)_2_ by varying the order of addition of reactants: (ii) Li_2_CO_3_ + Ca­(OH)_2_ + Ca­(OH)_2_ + Li_2_CO_3_, (iii) Ca­(OH)_2_ + Li_2_CO_3_ + Ca­(OH)_2_ + Li_2_CO_3_, and (iv) Ca­(OH)_2_ + Li_2_CO_3_ + Li_2_CO_3_ + Ca­(OH)_2_, the values of [OH^–^]_T_ are (i) 1.48, (ii) 1.47, (iii) 1.48, (iv) 1.48 mol L^–1^. That is, [OH^–^]_T_ is invariant of the order of mixing the reactants. As such, the causticization reaction is a true equilibrium process.

Finally, Figure 4b shows that the corresponding XRD traces of the one-step and three-step processes are largely similar, with one notable difference. Specifically, the most intense peak belongs to Ca­(OH)_2_ for the one-step, whereas it belongs to Li_2_CO_3_ for the three-step reaction. Since [OH^–^]_T_, and hence the conversions are the same for both processes, the difference arises from variations of primary crystallite sizes. The reaction time increased from 2 to 4.33 h for the three-step reaction, hence the ripening processes of the particles are likely to be different, resulting in larger crystallites and sharper peaks for Li_2_CO_3_, as predicted by the Scherrer equation.^45^

### The Backward Process

This Section discusses reverse causticization, the reaction between CaCO_3_ and concentrated LiOH solutions, which offers insight into the overall equilibrium. Reactions were initiated by mixing 4.17 mol L^–1^ LiOH with 10.8 g CaCO_3_ (or a reaction product), equivalent to complete conversion (*X* = 100%, forward process) at *c*_Li^+^,0_ = 30 g L^–1^. Five solids were tested as reactant: (1) synthesis product of the forward causticization reaction at *c*_Li^+^,0_ = 30 g L^–1^ and φ = 100% (*t*_reaction_ = 2 h), with major solid phases being Li_2_CO_3_ and Ca­(OH)_2_ (*X* = 35%, Figure 1d); (2) synthesis product at *c*_Li^+^,0_ = 10 g L^–1^ and φ = 100% (*t*_reaction_ = 2 h), dominated by calcite (*X* = 88%, Figure 1d); (3) CaCO_3_ (calcite); CaCO_3_ (calcite marble); and (5) as-prepared CaCO_3_ made by mixing equimolar CaCl_2_ and Na_2_CO_3_ solutions. These solids will be referred to as SP#1, SP#2, calcite, marble, and AP. As for AP, three batches were prepared for statistical relevance. The diffractogram of an AP is seen in Figure 5b; those of SP#1, SP#2, calcite and marble are seen in Figure S6a, Supporting Note 9.

**Figure 5:**
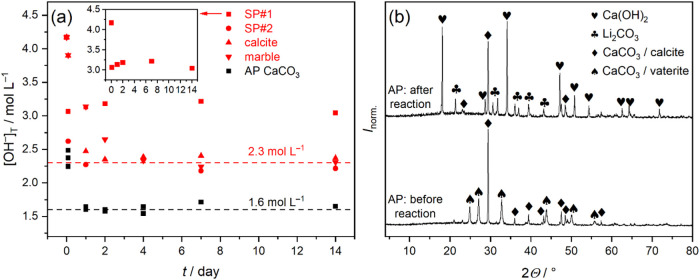
(a) Variation of the total concentration of OH^–^, [OH^–^]_T_, as a function of reaction time as obtained by reacting 4.17 mol L^–1^ LiOH solutions with different sources of CaCO_3_ (backward causticization reaction, *T* = 30 °C). For the latter, five different reactants were applied: synthesis product #1 obtained by reacting Li_2_CO_3_ with Ca­(OH)_2_ (SP#1; *c*_Li^+^,0_ = 30 g L^–1^, φ = 100%); synthesis product #2 (SP#2, *c*_Li^+^,0_ = 10 g L^–1^, φ = 100%); commercially available calcite and marble; as-prepared (AP) CaCO_3_ obtained by mixing Na_2_CO_3_ and CaCl_2_ solutions of equimolar concentration. The inset shows only reactions with SP#1. (b) Powder X-ray diffractograms of solids corresponding to the reaction with as-prepared CaCO_3_, before and after reaction reactions (*t*_reaction_ = 2 weeks). Symbols represent Ca­(OH)_2_ (PDF #84–1264), Li_2_CO_3_ (PDF #83–1454), calcite (PDF #83–1762) and vaterite (PDF #72–0506) polymorph of CaCO_3_.^35^ The XRD data were normalized such that the maximum intensity within each data set is 1.

Reaction times ranged from 2 h to 2 weeks. [OH^–^]_T_ (and [CO_3_^2–^]_T_) were measured and forming solids were analyzed before and after reaction; see Table S4 in Supporting Note 9. Figure 5a shows [OH^–^]_T_ over time for solids (1)–(5). For SP#1, [OH^–^]_T_ drops rapidly to ∼3.1 mol L^–1^ within 2 h and remains constant for longer reaction times. Corresponding diffractogram (Figure S6b) confirm the absence of CaCO_3_, suggesting a nearly quantitative reaction. (This is also in line with the high value of the relevant equilibrium constant *K*_–4_; see the discussion in Supporting Note 7). Conversely, SP#2, calcite and marble retain CaCO_3_ even after 2 weeks (Figure S6b), parallel to the formation of Li_2_CO_3_ and Ca­(OH)_2_. Independent of the initial solid, [OH^–^]_T_ stabilizes at ∼2.3 mol L^–1^ over time (Figure 5a, red horizontal line), markedly exceeding the ∼1.5 mol L^–1^ from the forward reaction (Figure 3a).

AP, however, yields a constant [OH^–^]_T_ of ∼1.6 mol L^–1^ (Figure 5a, black horizontal line), close to the plateau value of the forward process. This difference stems likely from the different compositions of the initial solids: calcite dominates SP#2, calcite, and marble, while AP contains significant amount of vaterite. Thus, two equilibrium concentrations, [LiOH]_eq_ = 1.6 and 2.3 mol L^–1^, correspond to vaterite and calcite polymorphs, respectively, which drive the reaction to different final states due to their different solubility. Although most of the vaterite in AP converts to calcite (Figures 5b), the persistence of [LiOH]_eq_ ≈ 1.6 mol L^–1^ after 2 weeks suggests that residual vaterite remains as minor component, likely as a surface phase undetectable by XRD.

In conclusion, the *K*_1_ equilibrium constant (eq 6) must be modified to account for the actual polymorph of CaCO_3_

10 $$K_{1}\left(\mathrm{calcite}\right)=\frac{K_{\mathrm{sp}}\left({\mathrm{Li}}_{2}{\mathrm{CO}}_{3}\right)\cdot K_{\mathrm{sp}}\left(\mathrm{Ca}{\left(\mathrm{OH}\right)}_{2}\right)}{K_{\mathrm{sp}}\left(\mathrm{Ca}{\mathrm{CO}}_{3},\mathrm{calcite}\right)}$$

11 $$K_{1}\left(\mathrm{vaterite}\right)=\frac{K_{\mathrm{sp}}\left({\mathrm{Li}}_{2}{\mathrm{CO}}_{3}\right)\cdot K_{\mathrm{sp}}\left(\mathrm{Ca}{\left(\mathrm{OH}\right)}_{2}\right)}{K_{\mathrm{sp}}\left(\mathrm{Ca}{\mathrm{CO}}_{3},\mathrm{vaterite}\right)}$$

Using literature values of the respective solubility products,^8,9,44,46^ i.e., *K*_sp_(Ca­(OH)_2_) = 5.6·10^–6^, *K*_sp_(Li_2_CO_3_) = 1.2·10^–3^, *K*_sp_(CaCO_3_, calcite) = 3.3·10^–9^, and *K*_sp_(CaCO_3_, vaterite) = 1.2·10^–8^, *K*_1_(calcite) = 2.0 and *K*_1_(vaterite) = 0.56 according to eq 6. It is worth mentioning that *K*_1_ corresponds to 25 °C, while experiments were performed at 30 °C. To study the possible effect of this minor difference, we estimated the temperature dependence of *K*_1_ using the enthalpies of the *K*_sp_ constants in the range of 5–75 °C.^9,44,47^ We show that the reaction is exothermic (contrasting a previous statement^3^) and that the enhancement of temperature from 25 to 30 °C lowers *K*_1_(vaterite) by 24%. More importantly, however, the decrease in *a*_±,LiOH_, which is directly related to [LiOH]_T_ (eq 8), is only 5%. Indeed, an independent experiment at 25 °C resulted in almost the same [OH^–^]_T_ (1.50 mol L^–1^) as the one at 30 °C (1.48 mol L^–1^) with *c*_Li^+^,0_ = 30 g L^–1^ at φ = 100%. For further details, see Figure S7 and discussion in Supporting Note 10.

The smaller value of *K*_1_(vaterite) stems from the better solubility of this polymorph, rendering its precipitation, i.e., product formation less favorable. Moreover, the notion that [LiOH]_T_ plateaus at 1.48 mol L^–1^ and *c*_Li^+^,0_ = 30 g L^–1^ (invariant of φ) after 2 h but increases to 2.0 mol L^–1^ after 2 weeks (Figure 2c), can be elucidated by simultaneous formation of vaterite and calcite. That is, first, vaterite forms under kinetic control, for which [LiOH]_eq_ is ∼1.6 mol L^–1^ (Figure 5a). This scheme is in fact supported by the observation that formation of vaterite is favored in moderately alkaline solutions (pH ≈ 10)^48^ by mixing Ca­(NO_3_)_2_ and NaHCO_3_ solutions. Further, freshly precipitating amorphous CaCO_3_, likely to occur during the forward reaction, first crystallizes to vaterite, governed by the dehydration of the amorphous phase.^49–51^ Applying longer reaction times, all vaterite present dissolves and reprecipitates as calcite under thermodynamic control, for which [LiOH]_eq_ is ∼2.3 mol L^–1^ (Figure 5a). Hence, 2.3 mol L^–1^ is expected to be the final equilibrium concentration also for AP which can only achieved by longer equilibration that may be impractical at an industrial scale.

The above hypothesis is verified in terms of mean activity coefficients of LiOH, γ_±,LiOH_. Specifically, the mean activity of LiOH, *a*_±,LiOH_ can be calculated from *K*_1_ (eq 8), while [LiOH]_eq_ can be estimated from the backward reaction (Figure 5a). In turn, these two quantities allow for the calculation of γ_±,LiOH_, which we compared with the data of Harned and Swindells, determined via concentration cell measurements of LiOH solutions;^33^ see Table 1. (For further details, see Figure S8 and further discussion in Supporting Note 11). We find that the calculated and literature values of γ_±,LiOH_ are in good agreement, the difference being only ∼5% for both calcite and vaterite.

**Table 1:** Equilibrium Constant of the Forward Causticization Reaction, *K*_1_, Equilibrium Concentration of LiOH, [LiOH]_eq_, Mean Activity and Activity Coefficient of LiOH, *a*_±,LiOH_ and *γ*_±,LiOH_, Assuming Amorphous Calcium Carbonate (ACC), Vaterite, or Calcite as Equilibrium Solid Phase, in Addition to Li_2_CO_3_ and Ca­(OH)_2_

	*K*_1_	[LiOH]_eq_/mol L^–1^	*a*_±,LiOH_	γ_±,LiOH_^calc^ ^a^	γ_±,LiOH_^ref^ ^b^
ACC	0.017	1.6	0.361	0.226	0.516
vaterite	0.56	1.6	0.865	0.541	0.516
calcite	2.0	2.3	1.19	0.517	0.492

a Calculated in this work from [LiOH]­eq and *a*_±,LiOH_ based on eq 8.

b Calculated from experimental data reported in ref 33.

Apart from vaterite, amorphous calcium carbonate^49–52^ needs to be considered as solubility-governing product for the forward process. Based on its solubility product at 25 °C, *K*_sp_(CaCO_3_, amorphous) = 4·10^–7^,^52^ the obtained γ_±,LiOH_ is less than half of the experimental one. The correct mean activity coefficient [LiOH]_eq_ = 1.6 mol L^–1^ can only be recovered if vaterite is the equilibrium-governing solid phase. Consequently, the two crystalline polymorphs, calcite and vaterite, are sufficient to explain the two equilibrium conditions with two distinct LiOH concentrations.

## Conclusions

In summary, we find that for dilute Li_2_CO_3_ suspensions (*c*_Li^+^,0_ = 5 and 10 g L^–1^), the carbonate salt is almost quantitatively converted to CaCO_3_ using Ca­(OH)_2_, regardless of the Ca­(OH)_2_:Li_2_CO_3_ molar ratio, φ. The high yield, *X* ≈ 90%, indicates that the reaction is entirely governed by the very low solubility of forming CaCO_3_ under these conditions. However, there are notable differences in terms of kinetics, since *X* first decreases, then increases upon increasing φ. This trend likely reflects a balance between common-ion effects (slowing down the reaction) and increasing surface of the reacting solids (accelerating the reaction).

The concentration of LiOH, [LiOH]_T_, is limited by an equilibrium value, [LiOH]_eq_, for concentrated suspensions (*c*_Li^+^,0_ = 15, 20, and 30 g L^–1^), due to the thermodynamic common-ion effect of LiOH: excess Li^+^/OH^–^ ions reduce the solubility of Li_2_CO_3_ and Ca­(OH)_2_, until their dissolution rates match that of CaCO_3_. This state defines the equilibrium among the three solids. We estimate [LiOH]_eq_ to be 1.6 mol L^–1^ or 3.7 wt %, based on reacting concentrated LiOH with as-prepared CaCO_3_ (backward process). This value is close to 1.5 mol L^–1^ (3.4 wt %) observed after 2 h of reaction in this work and has also been reported in the literature.^1,24^

Remarkably, 2 weeks of equilibration with *c*_Li^+^,0_ = 30 g L^–1^ yields a much higher [LiOH]_T_ of 2.0 mol L^–1^, indicating a second equilibrium with [LiOH]_eq_ = 2.3 mol L^–1^ or 5.2 wt %, based on the backward process employing pure calcite as reactant. These two states with [LiOH]_eq_ = 1.6 and 2.3 mol L^–1^, respectively, can be elucidated in terms of different polymorphs of CaCO_3_: vaterite forms first under kinetic control (1.6 mol L^–1^), then transforms into the more stable calcite (2.3 mol L^–1^). Thus, the maximum concentration of LiOH achievable via causticization is 2.3 mol L^–1^, though it might be unattainable for industrially relevant time scales. At this concentration, the corresponding *c*_Li^+^,0_ is ∼15.9 g L^–1^, the true thermodynamic upper limit for complete Li_2_CO_3_ conversion.

This study highlights the critical role of vaterite in the mechanism and thermodynamics of Li_2_CO_3_ conversion. However, key questions remain to fully understand the underlying chemistry of this process: (1) how does vaterite and calcite formation affect the ions concentrations over time, (2) what is the extent of association between Li^+^ and CO_3_^2–^ ions, and (3) does an intermediate mixed solid phase form, and how does it influence causticization kinetics?

## Experimental Section

### Materials and Sample Preparation

All samples were prepared by using deionized water (Merck Millipore Synergy UV). Li_2_CO_3_, LiOH, Na_2_CO_3_, CaCl_2_ and CaCO_3_ (VWR Chemicals) were of analytical grade and were used as received. A second source of CaCO_3_, (a. r. grade marble, Reanal) was also used; the granular solid was pulverized in an agate mortar with a pestle for ca. 20 min. Ca­(OH)_2_ was supplied by Lhoist and its purity was checked by thermogravimetric analysis (type Discovery TGA 5500 by TA Instruments) and total carbon detection (type Multi N/C 3100 by Analytik Jena). The composition of the solid was 97.7% Ca­(OH)_2_, 1.4% CaCO_3_ and 0.9% hydrating H_2_O. Thus, weighed amounts of Ca­(OH)_2_ were corrected for these impurities. For titrations, ∼0.25 mol L^–1^ HCl solutions were prepared volumetrically from 37 wt % HCl (a.r. grade, VWR Chemicals) and were standardized against KHCO_3_ using bromocresol green.

### Causticization Reactions

The conversion of Li_2_CO_3_ to LiOH and its reverse reaction were studied in a custom-made thermostatic bath, able to host 15 samples simultaneously. All experiments were performed in PTFE screw-top holders, connected by plastic tubes to maintain an inert gas atmosphere. Here, N_2_ gas was passed through a CO_2_ trap (Merck), followed by a gas washing bottle containing water, ensuring the gas entering the headspace was CO_2_-free and saturated with water vapor to minimize evaporation. The bath was connected to an external chiller (Julabo) for temperature control. Most reactions were performed at 30 °C, but several runs were also carried out at 25, 50, and 75 °C. Water was placed between the wall of the bath and PTFE holder for better heat exchange. The stability of the target temperature was 30.0 ± 0.5 °C. The suspensions were stirred at ca. 600 rpm with the aid of a multiposition magnetic stirrer (VELP). For the forward process, Li_2_CO_3_ was added into water and was pre-equilibrated for 20 min before adding Ca­(OH)_2_. The reaction mixtures were filtered through 0.45 μm PES filters equipped with a CO_2_ trap using a vacuum filtration apparatus. The filter cakes were subsequently dried in a desiccator with an infrared lamp under N_2_ atmosphere.

### Liquid-Phase Analysis

The progress of the causticization reactions was monitored using conductometry in several instances. This method relies on the fact that as the reaction proceeds, the concentration of carbonate ions (CO_3_^2–^) decreases while the concentration of hydroxide ions (OH^–^) increases significantly. Since OH^–^ has a much higher molar conductivity than CO_3_^2–^, this results in a measurable increase in the overall conductance of the solution. Conductance was measured by a Jenway 3540 Combined Conductivity/pH Meter with a conductivity probe with cell constant of 0.97 cm^–1^, as determined by measuring the conductance of a 0.1 mol L^–1^ KCl solution (made from a.r. grade solid from Reanal) at 25 °C. Based on at least three parallel measurements, the repeatability of κ, corresponding to suspensions at 2 h of reaction, was smaller than ±1% (Supporting Note 1). All reactions were performed at (30.0 ± 0.5) °C under constant stirring and N_2_ atmosphere.

The concentrations of OH^–^ and CO_3_^2–^ ions in the filtrates from various reaction mixtures were determined using a two-step titration method with 0.25 mol L^–1^ HCl as the titrant. Each filtrate was brought to volume in a volumetric flask, and a 2 mol L^–1^ NaCl solution (prepared from analytical reagent-grade solid, VWR Chemicals) was added to achieve a final concentration of 1 mol L^–1^ in the titrand. Since the relevant p*K* of phenolphthalein, used as indicator in the first titration step, falls in the weakly basic regime, the first equivalence point gives [OH^–^]_T_ + [CO_3_^2–^]_T_. The use of NaCl helps bring the p*K* values of the indicator and HCO_3_^–^ (∼9.5)^42^ ion closer to each other. Next, methyl red indicator was added to the colorless solution and titrated until a (transition) dark-orange color appeared. Following boiling to remove excess dissolved CO_2_, this step was repeated until the appearance of red color. The two equivalence points allow for obtaining [OH^–^]_T_ + [CO_3_^2–^]_T_ separately. Data are listed in Tables S2 and S4 in Supporting Notes 3 and 9, respectively. Further, it is worth discussing the possible presence of HCO_3_^–^ ions in solution. The equilibrium constant describing the protonation of CO_3_^2–^ ions yielding HCO_3_^–^ ions is *K* ^H^ = 12.3·10^10^ or log *K* ^H^ = 10.36 at infinite dilution.^42^ As for the solution with the lowest [LiOH]_T_ = 0.176 mol L^–1^, where formation HCO_3_^–^ could be most relevant, pH > 13, for which ratio of the free ions concentrations, [HCO_3_^–^]/[CO_3_^2–^], is estimated to be <0.001, rendering bicarbonate ions negligible for the studied solutions.

The calculation of conversion yields *X* is not trivial, since as LiOH forms, the density increases from 0.997 g mL^–1^ for H_2_O to 1.035 g mL^–1^ for 1.5 mol L^–1^ LiOH.^34^ Thus, the volume of the liquid phase changes, and it cannot be recovered after the reaction as the filter cake retains large amounts of solution despite using vacuum filtration. Therefore, *X* was obtained by dividing the actual [LiOH]_T_ (from titrations) by the maximum amount of LiOH, [LiOH]_T,max_, corresponding to *X* = 100% (eq 2). [LiOH]_T,max_ was calculated from the density-molality relationship reported earlier;^34^ further details on these calculations are provided in Supporting Note 3. In each case, the conversion was absolute conversion pertaining to the stoichiometrically limiting component (either Li_2_CO_3_ or Ca­(OH)_2_). Moreover, densities of solutions were measured by using a DMA 35 V. Three Basic vibrating-tube densitometer (Anton Paar); these data are listed in Tables S2 and S4 in Supporting Notes 3 and 9, respectively.

Further, [Li^+^]_T_ and [Ca^2+^]_T_ were measured for selected samples to check if Ca^2+^ contributed significantly to the solution composition. Metal-ion concentrations were obtained by monitoring the signal of the ^7^Li and ^44^Ca isotopes using an Agilent 7900 ICP-MS spectrometer. Here, samples were acidified by cc. HNO_3_ (1 wt % in the samples, NORMATOM, VWR Chemicals) and a solution containing yttrium was added as internal standard (0.1 ppm in the samples, ARISTAR, VWR Chemicals). Actual values of [Li^+^]_T_ and [Ca^2+^]_T_ and further discussion are given in Table S2 in Supporting Note 2.

### Solid-Phase Analysis

Dried solids obtained from the causticization reactions were analyzed via powder XRD, using a Rigaku Miniflex II diffractometer. Diffractograms were taken in the 2Θ = 4–100° range, using the *K*_α_ radiation of a Co source (λ = 1.7902 Å). The acquisition rate was 4° min^–1^. The thus obtained diffractograms were converted to the wavelength corresponding to Cu (1.5406°) applying the Bragg equation. Starting materials and reaction products were compared to literature references listed in the International Centre for Diffraction Data (ICDD).^35^

## Supplementary Material



## Notes

Conductivity and X-ray diffraction reproducibility measurements; tables listing LiOH yields and enhancement factors, solution densities, measured concentrations of OH^–^, CO_3_^2–^, and Ca^2+^ ions; conductivity dilution experiments; reaction products before and after reacting with LiOH for the backward reaction; discussion on the enthalpy of the causticization reaction; fitting the experimental LiOH mean activity coefficients; derivation of the equilibrium constants and mean activity of LiOH in equilibrium (PDF)
