# Supplementary material for: Lithium Carbonate Conversion to Lithium Hydroxide Using Calcium Hydroxide: Equilibrium is Governed by Vaterite Formation
Source: Inorg Chem. 2025 Nov 21;64(48):23575–85. doi: 10.1021/acs.inorgchem.5c04057 (PMC12690588; doi:10.1021/acs.inorgchem.5c04057)
Supplement: Supplementary file 1 [file ic5c04057_si_001.pdf]

Supplementary Information

Lithium carbonate conversion to lithium hydroxide using calcium hydroxide: Equilibrium is governed by vaterite formation

Péter Török[a], Ilona Halasiné-Varga[a], Laurent Duvivier[b], Maha Maimi[b], Olivier Hubert[b], Susanna Minnaar[b], Pál Sipos[a], Chris du Plessis[b], Bence Kutus[a*]

[a]*Department of Molecular and Analytical Chemistry, University of Szeged, Dóm tér 7-8, Szeged, H-6720 Hungary*
[b]*Lhoist, Business Innovation Centre, Rue de l'Industrie 31, 1400 Nivelles, Belgium**

Corresponding author. *E-mail address*: kutusb@chem.u-szeged.hu

1. Reproducibility of the conversion process probed by conductivity and XRD

The reproducibility of the conversion reaction was probed by conductivity as a function of reaction time for three independent measurements with initial Li^+ concentration, $c_{Li^+,0} = 10$ g L^{-1} and different $Ca(OH)_2$:Li_2CO_3 molar ratios, $\varphi = 50$, 100, 100%; see Figures S1–S3. Expectedly, larger scattering in the data is observed at $t_{reaction} < 100$ s, where the efficiency of mixing of reactants is most critical. Likewise, the uncertainty of data increases also with φ, since the amount of $Ca(OH)_2$ is twice and four times as much ($\varphi = 100\%$ and 200%) as that for $\varphi = 50\%$. Nevertheless, the curves exhibit an overall good reproducibility, particularly at the end of the reaction, where the relative standard deviations of κ at $t_{reaction} = 2$ h are as follows: 0.2% ($\varphi = 50\%$), 0.6% ($\varphi = 100\%$), and 0.8% ($\varphi = 200\%$).

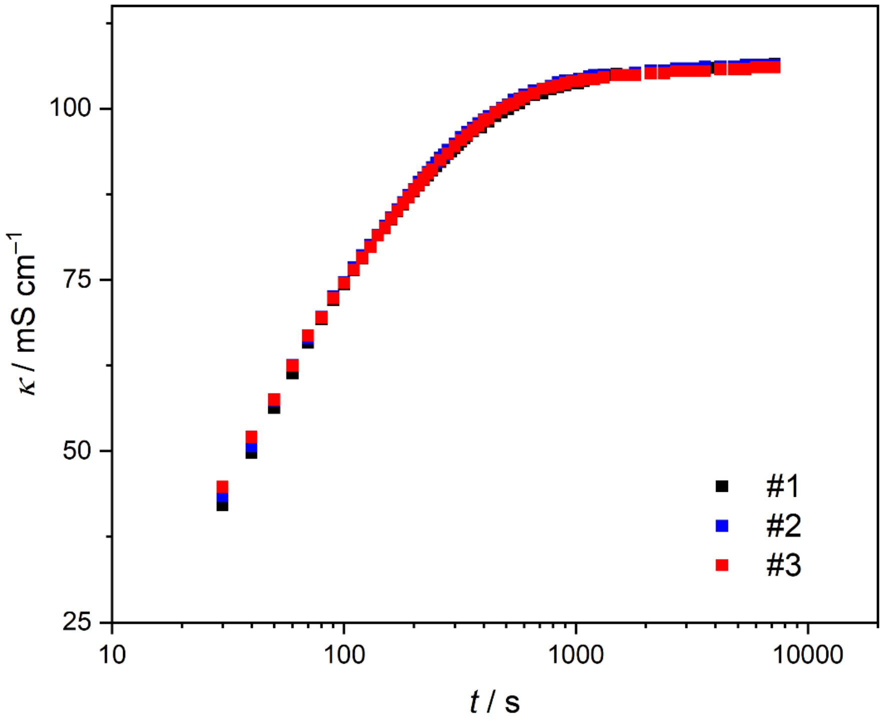

Figure S1 Variation of conductivity, κ, as a function time after addition of $Ca(OH)_2$ ($t = 0$) to a Li_2CO_3 suspension with initial Li^+ concentration of 10 g L^{-1} at $T = 30$°C. The $Ca(OH)_2$:Li_2CO_3 molar ratio was 50%. Data from three independent experiments are shown.

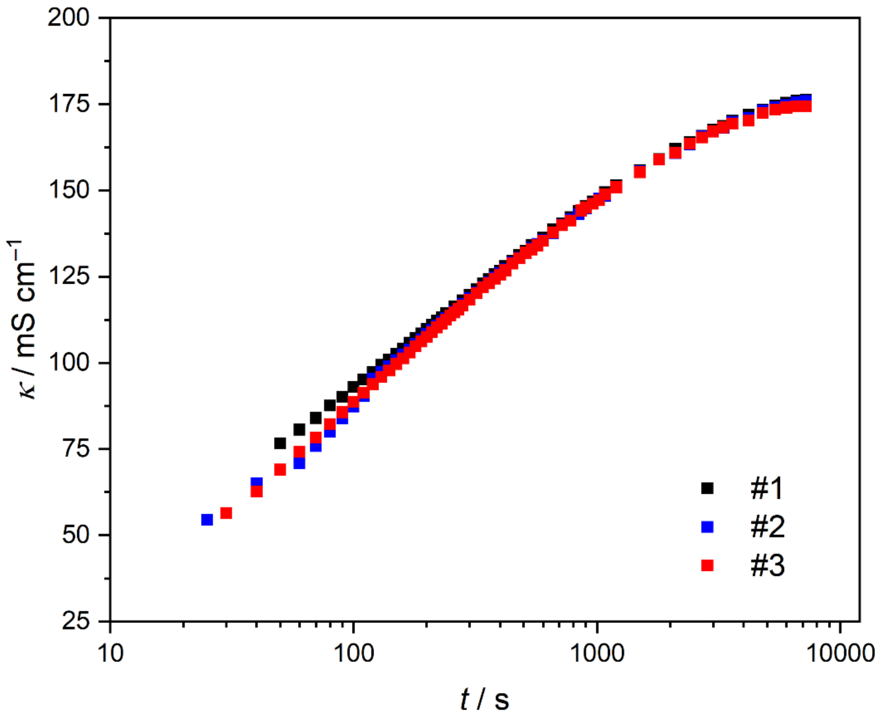

Figure S2 Variation of conductivity, κ, as a function time after addition of $Ca(OH)_2$ (t = 0) to a Li_2CO_3 suspension with initial Li^+ concentration of 10 g L^{-1} at T = 30°C. The $Ca(OH)_2$:Li_2CO_3 molar ratio was 100%. Data from three independent experiments are shown.

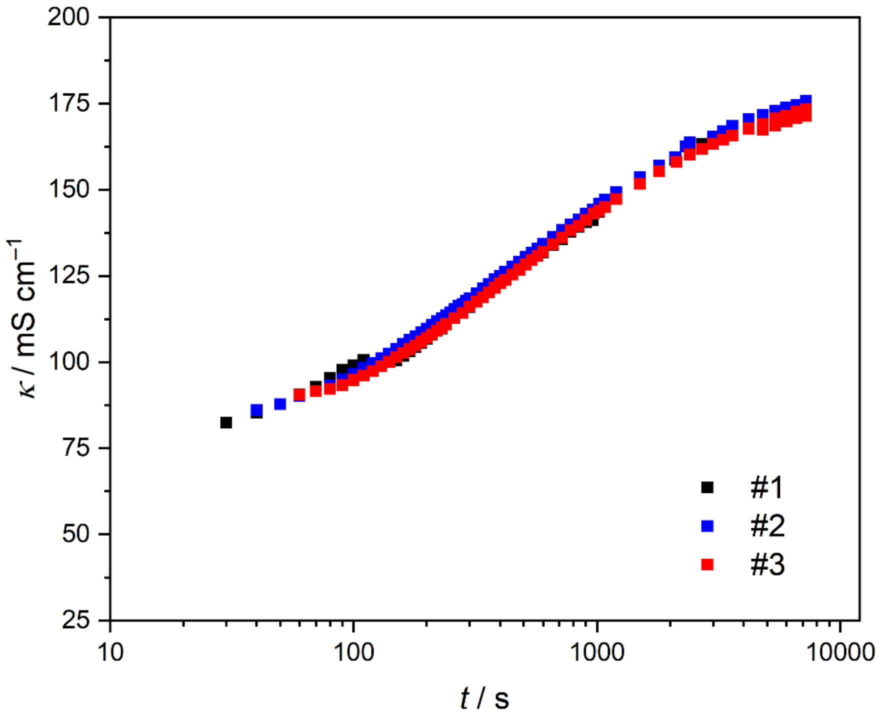

Figure S3 Variation of conductivity, κ, as a function time after addition of $Ca(OH)_2$ (t = 0) to a Li_2CO_3 suspension with initial Li^+ concentration of 10 g L^{-1} at T = 30°C. The $Ca(OH)_2$:Li_2CO_3 molar ratio was 200%. Data from three independent experiments are shown.

Likewise, the X-ray diffraction patterns of three solids obtained from independent experiments ($c_{Li^+,0}$ = 10 g L^{-1}, $t_{reaction}$ = 2 h), depicted in Figure S4. In each case, the dominant product is calcite with minor contributions from starting materials $Ca(OH)_2$ and Li_2CO_3. This agrees with the ~90% yield as determined by acid-base titrations (see Figure 2a, main text).

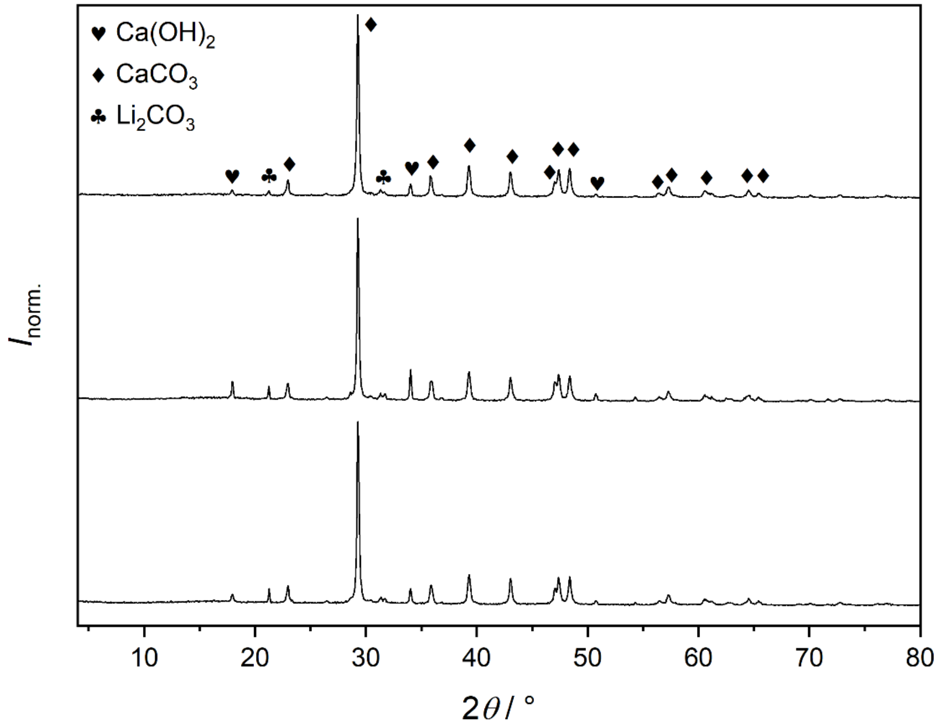

Figure S4 Powder X-ray diffractograms of solids obtained by reacting Li_2CO_3 suspensions ($c_{Li^+,0}$ = 10 g L^{-1}) with equimolar $Ca(OH)_2$ ($t_{reaction}$ = 2 h, T = 30°C). Diffraction patterns correspond to three independent experiments. Symbols represent $Ca(OH)_2$ (PDF #84–1264), Li_2CO_3 (PDF #83–1454) and product $CaCO_3$ (calcite polymorph, PDF #83–1762 [S1]). Data were normalized such that the maximum intensity within each dataset is 1.

2. Comparison of total concentrations for time-dependent experiments

Table S1 contains the results of time-dependent experiments for $c_{Li^+,0}$ = 10 g L^{-1} with reaction times ranging from 1 to 4 h. The total concentrations of OH^- and CO_3^{2-} ions in the supernatant, $[OH^-]_T$ and $[CO_3^{2-}]_T$, were obtained via two-step titrations, whereas $[Li^+]_T$ and $[Ca^{2+}]_T$ were measured via ICP-MS. The charge balance for all ions is the following:

$$[Li^+]_T + 2[Ca^{2+}]_T = [OH^-]_T + 2[CO_3{}^{2-}]_T \quad (S1)$$

Data indicate that the amount of Ca^{2+} ions is less than 0.02% of that of Li^+ ions. Hence, $[Li^+]_T = [OH^-]_T + 2[CO_3^{2-}]_T$, which means that $[Li^+]_T$ can be calculated from the anions concentrations. Indeed, $[OH^-]_T + 2[CO_3^{2-}]_T$ matches very well with $[Li^+]_T$ determined by ICP. (The highest

difference is 4.6% which is still acceptable considering the ±5% uncertainty of the data, particularly for a light isotope.)

In addition, values of $[OH^-]_T$ show that there is a considerable increase in the production of LiOH (1.18 to 1.25 mol L^{-1}) when increasing the reaction time from 1 to 2 hours. Extending the reaction time to 4 hours, however, gives rise only to a minor increase (1.25 to 1.26 mol L^{-1}). This is fully consistent with qualitative observations of the conductivity change as a function of time; see the discussion in the main text). Therefore, we chose 2 hours as the optimum time-window for the conversion reaction.

Table S1 Total concentrations of OH^-, CO_3^{2-}, Li^+ and Ca^{2+} ions, $[OH^-]_T$, $[CO_3^{2-}]_T$, $[Li^+]_T$, $[Ca^{2+}]_T$, obtained at different reaction times for the forward conversion reaction at 10 g L^{-1} initial concentration Li^+ ions. In addition, the difference between $[Li^+]_T$ and $[OH^-]_T + 2[CO_3^{2-}]_T$ is given. φ denotes the applied $Ca(OH)_2$:Li_2CO_3 molar ratio.

φ / %	$t_{reaction}$ / h	$[OH^-]_T$ / mol L^{-1} [a]	$[CO_3^{2-}]_T$ / mol L^{-1} [a]	$[OH^-]_T + 2[CO_3^{2-}]_T$ / mol L^{-1} [a]	$[Li^+]_T$ / mol L^{-1} [b]	diff. / %	$[Ca^{2+}]_T$ / mmol L^{-1} [b]
97.1	1	1.175	0.039	1.253	1.311	4.6	0.192
97.5	2	1.247	0.035	1.317	1.329	0.9	0.180
97.7	3	1.246	0.040	1.326	1.329	0.2	0.244
97.7	4	1.256	0.038	1.332	1.378	3.5	0.237

[a] Obtained from acid-base titrations.
[b] Obtained via ICP-MS measurements.

3. Calculation of the conversion yield

From a practical point of view, the extent of conversion or conversion yield, X, should be expressed as the percentage of formed and maximum amount of LiOH in terms of extensive variables, i.e. mass or amount of substance of the product. Since the experimentally most easily accessible variable is molar concentration (an intensive variable), calculation of X would require the exact volume or exact mass and density of the solution phase after filtration. This is difficult, since the filter cake always retains more or less liquid. Hence, we chose to define X as the ratio of total concentrations of formed and maximum amount of LiOH, $[LiOH]_T$ and $[LiOH]_{T,max}$. The total concentration of Ca^{2+} ions is negligible (see Table S1), hence there is no OH^- associated with Ca^{2+} in solution. Consequently, $[LiOH]_T = [OH^-]_T$ and $[LiOH]_{T,max} = [OH^-]_{T,max}$.

$$X = \frac{[LiOH]_T}{[LiOH]_{T,max}} = \frac{[OH^-]_T}{[OH^-]_{T,max}} \quad (S2)$$

Here, $[LiOH]_{T,max}$ (mol L^{-1}) represents an ideal state where there are only Li^+ and OH^- ions in solution. This state can actually never be reached, since some CO_3^{2-} ions always remain, due to the equilibrium solubility of $CaCO_3$ and/or possible ion-pairing between Li^+ and CO_3^{2-} ions, inferred

from the increase in $[CO_3^{2-}]_T$ at high LiOH concentration; see Figure 3b in the main text. In conclusion, any non-precipitated carbonate counts as unreacted Li_2CO_3, i.e. $X < 100\%$.

To calculate $[LiOH]_{T,max}$, not only the reaction, but also the changes in density, ρ, must be taken into account. Density calculations require the molality of the solution, b_{LiOH} (mol kg^{-1} solvent):

$$b_{\mathrm{LiOH}} = \frac{n_{\mathrm{LiOH,max}}}{m_{\mathrm{water}}} \tag{S3}$$

The maximum amount of LiOH, $n_{LiOH,max}$, can be obtained from the limiting component, which is $Ca(OH)_2$ in case of Li_2CO_3 excess, and Li_2CO_3 in case of $Ca(OH)_2$ excess. (For Li_2CO_3 excess, the actual $n_{LiOH,max}$ is smaller than the nominal one by 2.3%, due to the presence of 0.9% hydration water and 1.4% $CaCO_3$ in $Ca(OH)_2$.) The mass of solvent water is obtained as the sum of its initial amount and the 0.9% hydration water present in $Ca(OH)_2$. Next, the density can be obtained from the polynomial function as a function of b and T (in °C), reported in Ref. [S2]. Our calculations refer to 25°C, since densities of the solution phases were measured at 25°C.

Having ρ obtained, we calculate the total mass of the solution after reaction, m_T:

$$m_{\mathrm{T}} = m_{\mathrm{water}} + m_{\mathrm{Li_2CO_3}} + m_{\mathrm{Ca(OH)_2}} - m_{\mathrm{CaCO_3}} \tag{S4}$$

If either reactant is in excess, this excess amount is considered unreacted during the reaction; therefore, it does not contribute to m_T. Likewise, the 1.7% $CaCO_3$ contamination in $Ca(OH)_2$ counts as non-dissolving solid, too. Using m_T and ρ, the total volume, V_T, is readily obtained. Finally, $[LiOH]_{T,max}$ can be expressed as:

$$[\mathrm{LiOH}]_{\mathrm{T,max}} = \frac{n_{\mathrm{LiOH,max}}}{V_{\mathrm{T}}} \tag{S5}$$

All relevant experimental data for the forward ($Li_2CO_3 + Ca(OH)_2 \rightarrow 2LiOH + CaCO_3$) causticization reaction are listed in Table S2.

Table S2 Maximum and measured **t**otal concentrations of OH^-, $[OH^-]_{T,max}$ and $[OH^-]_T$; conversion yield, X, total concentrations of CO_3^{2-}, $[CO_3^{2-}]_T$; $[CO_3^{2-}]_T/[OH^-]_T$ ratios, and experimental densities, ρ, at different initial Li^+ concentrations, c_{Li^+}, and $Ca(OH)_2$:Li_2CO_3 molar ratios, φ, for the forward causticization. Unless otherwise stated, data pertain to $T = 30°C$ and $t_{reaction} = 2$ h. Values in parentheses indicate standard deviations of at least three parallel experiments.

$c_{Li,0^+}$ / g L^{-1}	φ / %	$[OH^-]_{T,max}$ / mol L^{-1}	$[OH^-]_T$ / mol L^{-1}	X / %	$[CO_3^{2-}]_T$ / mol L^{-1}	$[CO_3^{2-}]_T/[OH^-]_T$ / %	ρ / g mL^{-3}
5	24.4	0.176	0.166(3)	94(3)	0.107(2)	64(3)	1.0118(1)
5	48.8	0.353	0.331(1)	93.7(4)	0.083(2)	25.1(5)	1.0141(3)
5	73.3	0.529	0.493(2)	93.2(4)	0.066(1)	13.4(3)	1.0164(2)
5	97.6	0.705	0.646(4)	91.6(6)	0.024(1)	3.7(2)	1.0173(1)
5	146.4	0.722	0.679(3)	94.0(5)	0.008(1)	1.2(2)	1.0172(1)
5	195.8	0.723	0.681(3)	94.4(4)	0.008(1)	1.2(2)	1.0173(1)
10	24.5	0.353	0.327(7)	93(2)	0.084(4)	26(2)	1.0136(1)
10	48.8	0.705	0.642(3)	91.1(4)	0.050(1)	7.9(2)	1.0195(5)
10	73.3	1.059	0.958(3)	90.5(3)	0.0419(3)	4.37(5)	1.0267(2)
10	97.6	1.410	1.246(1)	88.4(2)	0.031(4)	2.4(3)	1.0326(1)
10[a]	97.7	1.411	1.353(8)	95.2(8)	0.034(6)	2.5(4)	1.0360(1)
10	146.4	1.445	1.30(1)	89.7(7)	0.032(8)	2.4(7)	1.0343(9)
10	195.3	1.444	1.304(1)	90.3(8)	0.024(1)	1.85(7)	1.0334(8)
15	24.4	0.529	0.50(1)	95(2)	0.060(5)	12(1)	
15	48.8	1.057	0.961(5)	90.8(5)	0.040(1)	4.13(9)	
15	73.2	1.586	1.329(6)	83.8(4)	0.034(2)	2.6(2)	
15	97.7	2.114	1.364(6)	64.5(3)	0.036(3)	2.6(2)	
15	146.5	2.164	1.392(2)	64.3(1)	0.037(2)	2.7(1)	
15	195.3	2.163	1.403(9)	64.9(4)	0.034(1)	2.5(1)	
20	24.4	0.758	0.654(1)	92.7(4)	0.054(1)	8.3(2)	1.0200(1)
20	48.9	1.412	1.248(4)	88.4(3)	0.038(1)	2.5(1)	1.0340(1)
20	73.3	2.117	1.426(4)	67.3(2)	0.041(3)	2.8(2)	1.0382(2)
20	97.7	2.818	1.444(6)	51.2(2)	0.036(2)	2.5(1)	1.0386(1)
20	146.5	2.851	1.48(2)	52.0(7)	0.034(5)	2.3(4)	1.0396(3)
20	195.8	2.852	1.46(1)	51.2(4)	0.035(3)	2.4(2)	1.0389(2)
30	24.5	1.062	0.972(3)	91.5(3)	0.041(2)	4.3(2)	1.0265(1)
30	48.9	2.116	1.47(1)	69.3(6)	0.032(4)	2.2(3)	1.0386(2)
30	73.1	3.167	1.474(3)	46.6(1)	0.031(3)	2.1(2)	1.0388(2)
30	97.6	4.206	1.477(5)	35.1(1)	0.031(3)	2.1(2)	1.0386(3)
30[a]	97.7	4.210	1.99(2)	47.2(5)	0.035(6)	1.8(3)	1.0509(3)
30[b]	97.7	4.210	1.499(9)	35.6(2)	0.030(2)	2.0(1)	1.0396(2)
30	146.6	4.305	1.50(1)	34.7(2)	0.032(1)	2.12(4)	1.0393(4)
30	195.4	4.303	1.48(1)	34.4(3)	0.039(4)	2.6(3)	1.0393(4)

[a] Reaction time was 2 weeks.
[b] Reaction temperature was 25°C.

4. Extent of conversion based on enhancement factors

Beside actual conversions, it is instructive to assess reaction efficiencies also in terms of the so-called enhancement factors, which quantify the increase in $[OH^-]_T$ in Figure 3a (main text). For short equilibration times, our findings strongly suggest that vaterite is the equilibrium-governing polymorph of calcium carbonate. Under these conditions, until $[LiOH]_T$ reaches ~1.5 mol L^{-1}, the reaction progresses nearly quantitatively. In turn, full conversion means that relative to $\varphi = 25\%$,

addition of two-, three- and fourfold $Ca(OH)_2$ to Li_2CO_3 (φ = 50, 75, and 100%) gives rise to a two-, three-, and fourfold increase in $[LiOH]_T$, which remains constant above φ = 100%.

Therefore, we compare the maximum and measured enhancement factors, f_{max} and f_{meas}, both normalized to their values at φ = 25%:

$$f_{max} = \frac{[LiOH]_{T,max,\varphi}}{[LiOH]_{T,max,\varphi=25\%}} \tag{S6}$$

$$f_{meas} = \frac{[LiOH]_{T,meas,\varphi}}{[LiOH]_{Tmeas,\varphi=25\%}} \tag{S7}$$

where $[LiOH]_{T,max,\varphi}$ is equal to $[LiOH]_T$ in the (theoretical) case of maximum conversion (X = 100%) at a given value of φ. Note that from the least (φ = 25%) to the most concentrated (φ = 200%) suspension, the density increases by 0.5%, 2.0%, 1.9%, and 1.2% at $c_{Li^+,0}$ = 5, 10, 20, and 30 g L^{-1}, respectively (Table S2). Since density changes have been taken into account for the calculation of $[LiOH]_{T,max}$, they are also included in f_{max}. Further, $f_{meas} = f_{max}$ in case of X = 100%.

The values of f_{max} and f_{meas} are listed in Table S3 and show good agreement for all φ at $c_{Li^+,0}$ = 5 and 10 g L^{-1}, and for φ = 50% at $c_{Li^+,0}$ = 15 and 20 g L^{-1}, indicating an almost quantitative reaction ($X \approx$ 90% or higher). However, f_{meas} differs significantly from f_{max} at φ > 50% ($c_{Li^+,0}$ = 15 and 20 g L^{-1}) and $\varphi \geq$ 25% ($c_{Li^+,0}$ = 30 g L^{-1}), signalling that the limiting $[LiOH]_{eq}$ is (almost) reached, pertaining to the state where all three solid phases are present.

Table S3 Calculated and measured enhancement factors, f_{max} and f_{meas} at different loadings of Li_2CO_3, $c_{Li^+,0}$, and different $Ca(OH)_2$:Li_2CO_3 molar ratios, φ.

$c_{Li^+,0}$ / g L^{-1}	5		10		15		20		30	
φ / %[a]	f_{max}	f_{meas}	f_{max}	f_{meas}	f_{max}	f_{meas}	f_{max}	f_{meas}	f_{max}	f_{meas}
50	2.00	1.99	2.00	1.96	2.00	1.91	2.00	1.91	1.99	1.51
75	3.00	2.97	3.00	2.93	3.00	2.65	3.00	2.18	2.98	1.52
100	4.00	3.89	4.00	3.81	4.00	2.72	3.99	2.21	3.96	1.52
150	4.10	4.09	4.09	3.96	4.10	2.77	4.04	2.27	4.05	1.54
200	4.10	4.10	4.09	3.99	4.09	2.79	4.04	2.23	4.05	1.52

[a] Nominal values. Actual values (Table S2) are slightly smaller due to $CaCO_3$ and H_2O impurities in $Ca(OH)_2$ and deviations between weighed and calculated masses of the reactants.

5. Derivation of the equilibrium constant of the causticization reaction

Regarding the overall conversion reaction, the state of reactants and products must be set. For practical relevance, solid Li_2CO_3(s), $Li_2CO_{3(s)}$, solid $Ca(OH)_2$, $Ca(OH)_{2(s)}$, solid $CaCO_3$, $CaCO_{3(s)}$

(which may exist as different polymorphs), and dissolved LiOH, $LiOH_{(aq)}$, are considered in chemical equilibrium. The corresponding reaction reads as:

$$Li_2CO_{3(s)} + Ca(OH)_{2(s)} \rightleftharpoons 2Li^+_{(aq)} + 2OH^-_{(aq)} + CaCO_{3(s)} \tag{S8}$$

The corresponding equilibrium constant is denoted as K_1. For the sake of clarity, $LiOH_{(aq)}$ is considered as a fully dissociated electrolyte yielding Li^+ and OH^- ions. It is worth mentioning that ion association has been reported for concentrated LiOH solutions [S3,S4], which means that some of the free Li^+ and OH^- ions may form $LiOH^0$ ion-pairs.

The causticization reaction (Eq. S8) can be composed from three equilibrium processes, involving the dissolution of $Li_2CO_{3(s)}$, $Ca(OH)_{2(s)}$, and $CaCO_{3(s)}$:

$$Li_2CO_{3(s)} \rightleftharpoons 2Li^+_{(aq)} + CO_3{}^{2-}{}_{(aq)} \tag{S9}$$

$$Ca(OH)_{2(s)} \rightleftharpoons Ca^{2+}{}_{(aq)} + 2OH^-_{(aq)} \tag{S10}$$

$$CaCO_{3(s)} \rightleftharpoons Ca^{2+}{}_{(aq)} + CO_3{}^{2-}{}_{(aq)} \tag{S11}$$

The equilibrium state of the above reactions can be expressed in terms of solubility products (phases in the subscripts are omitted):

$$K_{\mathrm{sp}}(\mathrm{Li_2CO_3}) = \frac{(a_{\mathrm{Li^+}})^2 a_{\mathrm{CO_3{}^{2-}}}}{a_{(\mathrm{Li_2CO_3})}} \tag{S12}$$

$$K_{\mathrm{sp}}(\mathrm{Ca(OH)_2}) = \frac{a_{\mathrm{Ca^{2+}}}(a_{\mathrm{OH^-}})^2}{a_{(\mathrm{Ca(OH)_2})}} \tag{S13}$$

$$K_{\mathrm{sp}}(\mathrm{CaCO_3}) = \frac{a_{\mathrm{Ca^{2+}}} a_{\mathrm{CO_3{}^{2-}}}}{a_{(\mathrm{CaCO_3})}} \tag{S14}$$

where a_X are the thermodynamic activities for a given species X, including the solid phase itself. (Note that activities are dimensionless, so are solubility products.) As for the solid, $a = 1$ in thermodynamic equilibrium, $a > 1$ for a supersaturated, and $a < 1$ for an undersaturated solution with no solid present.

The above constants can be derived from the corresponding standard free Gibbs energy changes, $\Delta_r G^{\emptyset}$:

$$\Delta_r G_1{}^{\emptyset} = -RT \ln K_1 \tag{S15}$$

$$\Delta_r G_2{}^{\emptyset} = -RT \ln\ K_{\mathrm{sp}}(\mathrm{Li_2CO_3}) \tag{S16}$$

$$\Delta_r G_3{}^{\emptyset} = -RT \ln K_{\mathrm{sp}}(\mathrm{Ca(OH)_2}) \tag{S17}$$

$$\Delta_r G_4{}^{\emptyset} = -RT \ln K_{\mathrm{sp}}(\mathrm{CaCO_3}) \tag{S18}$$

The conversion reaction (1) can be obtained from the algebraic sum of reactions (2), (3), and (4):

$$(1) = (2) + (3) - (4) \tag{S19}$$

Hess's law states that the standard free energy of a chemical process is independent of the sequence of steps taken, which allows for the expression of $\Delta_r G_1{}^{\emptyset}$ by combining the other three standard Gibbs free energies, according to Eq. 20:

$$\Delta_r G_1{}^{\emptyset} = \Delta_r G_2{}^{\emptyset} + \Delta_r G_3{}^{\emptyset} - \Delta_r G_4{}^{\emptyset} \tag{S20}$$

Combining Eqs. S15–S18 and S20, K_1 is readily obtained:

$$\ln K_1 = \ln\ K_{\mathrm{sp}}(\mathrm{Li_2CO_3}) + \ln K_{\mathrm{sp}}(\mathrm{Ca(OH)_2}) - \ln K_{\mathrm{sp}}(\mathrm{CaCO_3}) \tag{S21}$$

or analogously:

$$K_1 = \frac{K_{\mathrm{sp}}(\mathrm{Li_2CO_3}) K_{\mathrm{sp}}(\mathrm{Ca(OH)_2})}{K_{\mathrm{sp}}(\mathrm{CaCO_3})} \tag{S22}$$

Finally, the K_{sp} constants can be substituted by the products of thermodynamic activities (Eqs. S12–14), noting the activity of each solid is in chemical equilibrium:

$$K_1 = \frac{(a_{\mathrm{Li^+}})^2 a_{\mathrm{CO_3{}^{2-}}} \cdot a_{\mathrm{Ca^{2+}}} (a_{\mathrm{OH^-}})^2}{a_{\mathrm{Ca^{2+}}} a_{\mathrm{CO_3{}^{2-}}}} = (a_{\mathrm{Li^+}} a_{\mathrm{OH^-}})^2 \tag{S23}$$

Based on the literature values of $K_{\mathrm{sp}}(\mathrm{Li_2CO_3}) = 1.2 \cdot 10^{-3}$ [S5,S6], $K_{\mathrm{sp}}(\mathrm{Ca(OH)_2}) = 5.6 \cdot 10^{-6}$ [S7], $K_{\mathrm{sp}}(\mathrm{CaCO_3}$, calcite$) = 3.3 \cdot 10^{-9}$, and $K_{\mathrm{sp}}(\mathrm{CaCO_3}$, vaterite$) = 1.2 \cdot 10^{-8}$ [S8], $K_1 = 2.0$ (calcite) or 0.56 (vaterite) at 25°C.

6. Relationship between the equilibrium constant and the mean ionic activity of LiOH

The activities $a_{\mathrm{Li^+}}$ and $a_{\mathrm{OH^-}}$ can be expressed with the equilibrium molar concentrations, $[\mathrm{Li^+}]_{\mathrm{eq}}$ $[\mathrm{OH^-}]_{\mathrm{eq}}$, and the activity coefficients, $\gamma_{\mathrm{Li^+}}$ and $\gamma_{\mathrm{OH^-}}$:

$$K_1 = \frac{(\gamma_{\mathrm{Li^+}} [\mathrm{Li^+}]_{\mathrm{eq}} \cdot \gamma_{\mathrm{OH^-}} [\mathrm{OH^-}]_{\mathrm{eq}})^2}{(c^{\emptyset})^4} \tag{S24}$$

where $c^{\emptyset}$ is the standard molar concentration (1 mol $\mathrm{L^{-1}}$), to ensure that a and K_1 remain dimensionless. Using the individual γ coefficients, the mean ionic activity coefficient of LiOH, $\gamma_{\pm,\mathrm{LiOH}}$, can be defined according to the Debye-Hückel theory [S9]:

$$\left(\gamma_{\pm,\mathrm{LiOH}}\right)^2 = \gamma_{\mathrm{Li^+}}\gamma_{\mathrm{OH^-}} \tag{S25}$$

$$K_1 = \frac{\left(\left(\gamma_{\pm,\mathrm{LiOH}}\right)^2[\mathrm{Li^+}]_{\mathrm{eq}}[\mathrm{OH^-}]_{\mathrm{eq}}\right)^2}{\left(c^{\emptyset}\right)^4} = \frac{\left(\gamma_{\pm,\mathrm{LiOH}}\right)^4\left([\mathrm{Li^+}]_{\mathrm{eq}}[\mathrm{OH^-}]_{\mathrm{eq}}\right)^2}{\left(c^{\emptyset}\right)^4} \tag{S26}$$

The above expression means that if K_1 and the exact values of $[Li^+]_{eq}$ and $[OH^-]_{eq}$ are known, $\gamma_{\pm,LiOH}$ can be calculated. Nevertheless, this equation can be further simplified if $[Li^+]_{eq} \approx [OH^-]_{eq}$, which means that $[Ca^{2+}]_{eq} << [Li^+]_{eq}$ and $[CO_3^{2-}] << [OH^-]_{eq}$. These conditions directly follow from the charge balance (Eq. S1).

ICP-MS measurements of $[Li^+]_T$ and $[Ca^{2+}]_T$ confirm that the first criterium is met (Table S1), whereas titration data (Table S2) show that the amount of CO_3^{2-} ions relative to OH^- ions fluctuate mainly between 1% and 3% at the two equilibrium LiOH concentrations, 1.6 and 2.3 mol L^{-1}. (The final equilibrium concentration depends on whether vaterite or calcite is the equilibrium solid.) Hence, $[Li^+]_{eq} \approx [OH^-]_{eq} \approx [LiOH]_{eq}$ applies if all three solids are present in equilibrium. (The validity of this relation relies on the assumption that ion-pairing is negligible; hence, the equilibrium concentrations of the free ions can be approximated by the equilibrium total concentration of LiOH.) Eq. S26 may now be rewritten as:

$$K_1 \approx \frac{\left(\gamma_{\pm,\mathrm{LiOH}}\right)^4\left([\mathrm{LiOH}]_{\mathrm{eq}}\right)^4}{\left(c^{\emptyset}\right)^4} = \left(\gamma_{\pm,\mathrm{LiOH}}\frac{[\mathrm{LiOH}]_{\mathrm{eq}}}{c^{\emptyset}}\right)^4 \tag{S27}$$

Note that the third term in Eq. S27 has the same form as that of the individual ions, i.e. $\gamma c/c^{\emptyset}$, from which a 'mean' activity for LiOH, $a_{\pm,LiOH}$, can be defined:

$$K_1 \approx \left(\gamma_{\pm,\mathrm{LiOH}}\frac{[\mathrm{LiOH}]_{\mathrm{eq}}}{c^{\emptyset}}\right)^4 \stackrel{\mathrm{def}}{=} \left(a_{\pm,\mathrm{LiOH}}\right)^4 \tag{S28}$$

7. Equilibria involving only two solid phases

Considering different equilibrium states, three further cases should be considered. If the suspension is concentrated for Li_2CO_3 but dilute for $Ca(OH)_2$, the latter dissolves completely upon reaction and only two solid phases remain in equilibrium; that is, Li_2CO_3 and $CaCO_3$, respectively. This is the case for systems with $c_{Li^+,0}$ = 5 and 10 g L^{-1} in case of Li_2CO_3 excess:

$$Li_2CO_{3(s)} + Ca^{2+}{}_{(aq)} + 2OH^-{}_{(aq)} \rightleftharpoons 2Li^+{}_{(aq)} + 2OH^-{}_{(aq)} + CaCO_{3(s)} \tag{S29}$$

which simplifies to:

$$Li_2CO_{3(s)} + Ca^{2+}{}_{(aq)} \rightleftharpoons 2Li^+{}_{(aq)} + CaCO_{3(s)} \tag{S30}$$

Using the same method as for K_1, the new equilibrium constant, K_2, can be obtained:

$$K_2 = \frac{K_{sp}(Li_2CO_3)}{K_{sp}(CaCO_3)} = \frac{K_1}{K_{sp}(Ca(OH)_2)} = \frac{(a_{Li^+})^2}{a_{Ca^{2+}}} \quad (S31)$$

Using the values of the thermodynamic solubility products, $K_2 = 3.6 \cdot 10^5$ (calcite) or $1.0 \cdot 10^5$ (vaterite). The large value of this constant translates to a virtually quantitative reaction under the conditions set by Eq. S30. The quantitative conversion of $Ca(OH)_2$ in case of Li_2CO_3 excess is supported by the high conversions at $c_{Li^+,0} = 5$ and 10 g L^{-1} and the absence of solid $Ca(OH)_2$ in the X-ray diffractogram ($X > 90\%$; Figures 2a and 2b, main text).

The second type of equilibrium pertains to using dilute Li_2CO_3 suspensions and $Ca(OH)_2$ excess:

$$2Li^+_{(aq)} + CO_3{}^{2-}{}_{(aq)} + Ca(OH)_{2(s)} \rightleftharpoons 2Li^+_{(aq)} + 2OH^-_{(aq)} + CaCO_{3(s)} \quad (S32)$$

which simplifies to:

$$CO_3{}^{2-}{}_{(aq)} + Ca(OH)_{2(s)} \rightleftharpoons 2OH^-_{(aq)} + CaCO_{3(s)} \quad (S33)$$

The equilibrium constant, K_3, reads as:

$$K_3 = \frac{K_{sp}(Ca(OH)_2)}{K_{sp}(CaCO_3)} = \frac{K_1}{K_{sp}(Li_2CO_3)} = \frac{(a_{OH^-})^2}{a_{CO_3{}^{2-}}} \quad (S34)$$

Based on the solubility products reported in literature [S5–S8], $K_3 = 1.7 \cdot 10^3$ (calcite) or $4.67 \cdot 10^2$ (vaterite), which are still large, consistent with the $X > 90\%$ conversions also for $Ca(OH)_2$ excess, and the absence of solid Li_2CO_3 in the powder diffractograms (Figures 2a and 2b, main text).

For both cases, if the concentration of the forming LiOH reaches a limiting value corresponding to $a_{\pm,LiOH}$ (Eq. S28), all three solid phases will be present in equilibrium.

The third equilibrium is important to elucidate the disappearance of $CaCO_3$ in the case of the reverse causticization reaction, where (4.17 mol L^{-1}) LiOH solution was mixed with a synthesis product of the forward process ($c_{Li^+,0} = 30$ g L^{-1}, $\varphi = 100\%$), in which the dominant phases are Li_2CO_3 and $Ca(OH)_2$. For this experiment, the two equilibrium phases were $Ca(OH)_2$ and Li_2CO_3. The corresponding reaction reads:

$$Li_2CO_{3(s)} + Ca(OH)_{2(s)} \rightleftharpoons 2Li^+_{(aq)} + 2OH^-_{(aq)} + Ca^{2+}_{(aq)} + CO_3{}^{2-}{}_{(aq)} \quad (S35)$$

The equilibrium constant, K_4, is the following:

$$K_4 = K_{sp}(Ca(OH)_2)K_{sp}(Li_2CO_3) = K_1 K_{sp}(CaCO_3) = (a_{Li^+}a_{OH^-})^2 a_{Ca^{2+}} a_{CO_3{}^{2-}} \quad (S36)$$

Accordingly, $K_4 = 6.7 \cdot 19^{-9}$, which means that the equilibrium constant of the reverse process, $K_{-4} = 1/K_4$, is very high ($1.5 \cdot 10^8$). That is, $CaCO_3$ dissolves completely upon reacting with LiOH, yielding $Ca(OH)_2$ and Li_2CO_3, until $a_{\pm,LiOH}$ is reached. At this point, undissolved $CaCO_3$ remains as a third equilibrium solid phase.

In summary, the driving force of the forward process with either two or three phases in equilibrium is the precipitation of $CaCO_3$, whereas the reverse reaction is driven by the precipitation of Li_2CO_3 and $Ca(OH)_2$. However, it cannot be judged which equilibrium will prevail based on solely on the equilibrium constants, because a solid (being reactant or product), might completely be consumed during reaction. Hence, the final state of the system depends not only on the equilibrium constants but also on the initial amount of the solids.

8. Dilution effects in conductivity

Addition of Li_2CO_3 to an equilibrated mixture of equimolar Li_2CO_3 and $Ca(OH)_2$ to increase $c_{Li^+,0}$ from 10 to 30 g L^{-1} gives rise to a decrease from 172 to 161 mS cm^{-1} (Figure 4a, main text). To confirm that this not due to further reaction between residual $Ca(OH)_2$ and the newly added lithium salt, we repeated the experiment but without any $Ca(OH)_2$ present. Figure S5a shows that the initial 17.2 mS cm^{-1} conductivity of an equilibrium Li_2CO_3 suspension ($c_{Li^+,0}$ = 10 g L^{-1}) drops to 16.4 mS cm^{-1} in ca. 30 minutes. Since more Li_2CO_3 cannot dissolve in an already equilibrated suspension, this decrease arises from by introducing insulating solid particles, which in turn lower the concentrating of conducting species [S10].

Addition of $Ca(OH)_2$ to have again φ = 100% results in an initial drop from 161 to 151 mS cm^{-1} (Figure 4a, main text). Repeating the experiment by adding the same amount of $Ca(OH)_2$ to a $Ca(OH)_2$ suspension with not Li_2CO_3 present also lowers conductivity from 8.7 to 8.1 mS cm^{-1} requiring the same time (Figure S5b).

In conclusion, the two conductivity drops observed for the forward reaction are associated with simple dilution effects. As the conductivities before solid addition in the Li_2CO_3 or $Ca(OH)_2$ control experiments (17.2 and 8.7 mS cm^{-1}) are much smaller than those with both solids present (172 and 161 mS cm^{-1}) the conductivity drops are also much smaller.

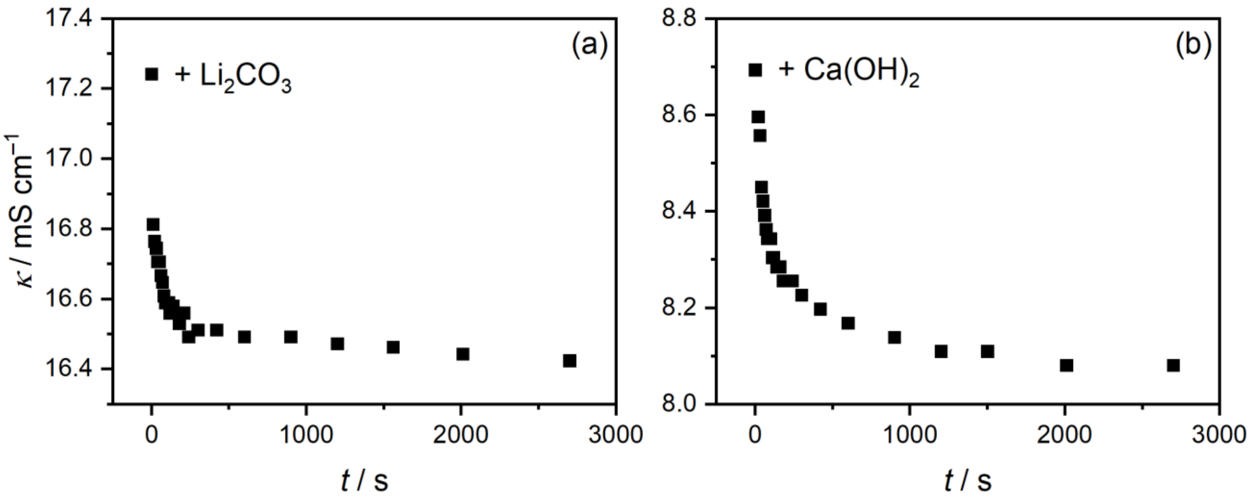

Figure S5 Conductivity changes over time upon adding **(a)** 5.34 g Li_2CO_3 to an equilibrated aqueous suspension containing 2.67 g Li_2CO_3 or **(b)** 5.34 $Ca(OH)_2$ to 2.67 g $Ca(OH)_2$. The amount of Li^+ before and after salt addition is 10 and 30 g L^{-1}, respectively, while the amounts of $Ca(OH)_2$ are equimolar.

9. Backward reaction: X-ray diffractograms and concentration data

Figure S6a shows the diffraction patterns of commercial marble and calcite, synthesis product #1 and #2 (SP#1 and SP#2), obtained from the forward process, i.e. $Li_2CO_3 + Ca(OH)_2 \rightarrow 2LiOH + CaCO_3$. The dominant phase in SP#1 is Li_2CO_3 and $Ca(OH)_2$, whereas $CaCO_3$ (calcite) is the major solid phase in SP#2. Reacting these solids with 4.17 mol L^{-1} LiOH solution for two weeks (backward reaction, i.e. $2LiOH + CaCO_3 \rightarrow Li_2CO_3 + Ca(OH)_2$) yields different solid phases, depicted in Figure S6b. In each case, products Li_2CO_3 and $Ca(OH)_2$ are formed, and $CaCO_3$ remains for commercial $CaCO_3$ and SP#2. However, this phase is absent in the case of SP#1, which already had smaller fraction of $CaCO_3$, suggesting that the solid is completely consumed after 2 weeks.

Data for $[OH^-]_T$, $[CO_3^{2-}]_T$, their ratios, and experimental densities as a function of reaction time are listed in Table S4.

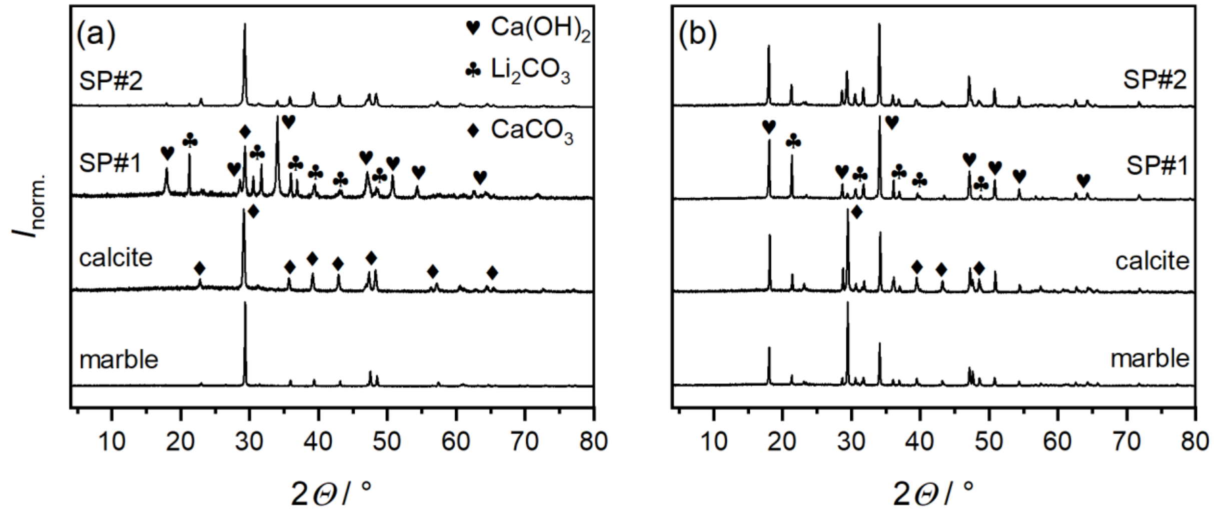

Figure S6 Powder X-ray diffractograms of solids used as reactant **(a)** before and **(b)** after reacting with 4.17 mol L^{-1} LiOH solution ($t_{reaction}$ = 2 weeks, T = 30°C). The reactants were commercial marble (calcite), calcite, synthesis product #1 (SP#1) obtained by reacting Li_2CO_3 with $Ca(OH)_2$ (SP#1; $c_{Li^+,0}$ = 30 g L^{-1}, φ = 100%), and synthesis product #2 (SP#2, $c_{Li^+,0}$ = 10 g L^{-1}, φ = 100%). Symbols represent $Ca(OH)_2$ (PDF #84–1264), Li_2CO_3 (PDF #83–1454), and calcite polymorph of $CaCO_3$ (PDF #83–1762) [S1]. The XRD data were normalized such that the maximum intensity within each dataset is 1.

Table S4 Measured **t**otal concentrations of OH^- CO_3^{2-} and $[OH^-]_T$ and $[CO_3^{2-}]_T$; $[CO_3^{2-}]_T/[OH^-]_T$ ratios, and experimental densities, ρ, using 4.17 mol L^{-1} LiOH solution and different sources of $CaCO_3$ as reactants (backward causticization reaction, T = 30°C).

source of $CaCO_3$	$t_{reaction}$ / h	$[OH^-]_T$ / mol L^{-1}	$[CO_3^{2-}]_T$ / mol L^{-1}	$[CO_3^{2-}]_T/[OH^-]_T$ / %	ρ / g mL^{-3}
	0	4.17			
calcite	2	3.90	0.079	2.0	1.0929
calcite	2	3.87	0.078	2.0	1.0937
calcite	24	2.47	0.054	2.2	1.0625
calcite	48	2.35	0.048	2.1	1.0599
calcite	96	2.39	0.038	1.6	1.0603
calcite	168	2.40	0.033	1.4	1.0594
calcite	336	2.37	0.031	1.3	1.0594
marble	0	4.17			
marble	2	3.90	0.058	1.5	1.0939
marble	24	3.14	0.045	1.4	1.0765
marble	48	2.65	0.036	1.4	1.0663
marble	96	2.33	0.036	1.5	1.0582
marble	168	2.24	0.029	1.3	1.0571
marble	336	2.31	0.027	1.2	1.0574
SP#1	0	4.17			
SP#1	2	3.06	0.035	1.2	1.0741
SP#1	24	3.11	0.029	0.9	1.0751
SP#1	48	3.16	0.024	0.8	1.0754
SP#1	168	3.22	0.020	0.6	1.0774
SP#1	336	3.04	0.035	1.2	1.0743
SP#2	0	4.17			
SP#2	2	2.58	0.040	1.6	1.0643
SP#2	24	2.24	0.026	1.1	1.0562
SP#2	168	2.18	0.033	1.5	1.0554
SP#2	336	2.18	0.035	1.6	1.0551
AP#1	0	4.17			
AP#1	2	2.37	0.058	2.4	1.0612
AP#1	24	1.61	0.046	2.9	1.0427
AP#1	48	1.58	0.036	2.3	1.0420
AP#1	96	1.54	0.041	2.7	1.0411
AP#2	0	4.17			
AP#2	2	2.25	0.053	2.4	1.0594
AP#2	24	1.65	0.038	2.3	1.0446
AP#2	48	1.60	0.035	2.2	1.0426
AP#2	168	1.71	0.028	1.6	1.0450
AP#3	0	4.17			
AP#3	2	2.49	0.063	2.5	1.0648
AP#3	96	1.65	0.033	2.0	1.0431
AP#3	336	1.65	0.037	2.2	1.0436

10. Temperature-dependence of the forward reaction

Literature states that the causticization reaction is endothermic [S11], i.e. K_1 increases with rising temperature. Consistent with this, X increased from 52.5 only to 59.5% upon raising the temperature from 25 to 100°C [S12]. To estimate the differences between 25°C and 30°C, we carried out additional experiments at $c_{Li^+,0}$ = 30 g L^{-1} at φ = 100% at 25°C, under the same experimental conditions. The thus obtained value of $[OH^-]_T$ is 1.50 mol L^{-1}, which is essentially the same as 1.48 mol L^{-1} obtained at 30°C, suggesting that there is no significant difference in the conversion efficiency in this narrow temperature window.

The temperature dependence of K_1 (or log K_1) can be calculated based on the temperature dependence of the individual K_{sp} solubility products. For $Ca(OH)_2$, we employed the van 't Hoff equation with $\Delta_r H_3^{ø}$ = –20 kJ mol^{-1} reported earlier for the temperature range of 5–75°C [S13], while for the other two solids, we applied the log $K_{sp}(T)$ functions obtained from non-linear fitting [S6,S8]. Since the measured $[LiOH]_T$ concentrations were obtained from the forward process, where vaterite has been suggested to be the equilibrium polymorph of $CaCO_3$, log K_{sp}($CaCO_3$, vaterite) was used to calculate K_1(vaterite) at different temperatures.

The calculated equilibrium constants are plotted as a function of reciprocal temperature in Figure S8. Surprisingly, the data show that the conversion reaction is exothermic since K_1(vaterite) decreases with increasing temperature. As for 25 and 30°C, K_1(vaterite) = 1.97 and 1.59, respectively. However, $a_{\pm,LiOH}$, which is directly related to the measured LiOH concentration, decreases from 0.855 to 0.816, which is ~5%. It is reasonable to assume that $\gamma_{\pm,LiOH}$ changes only marginally from 25 to 30°C, hence the similar $[LiOH]_{eq}$ concentrations at the two temperatures.

Nevertheless, the increase in X with increasing temperature from 25 to 60°C [S12] speaks against the exothermic nature of the causticization reaction. A plausible explanation is that the reaction was not in equilibrium at 25°C. Indeed, X of 52.5% increased to 64–68% upon increasing the reaction time from 1 to 10 hours, and we obtained also X = 64% at the same Li^+ concentration (15 g L^{-1}), although after 2 hours. Therefore, the higher conversion at 60°C is not the result of a higher equilibrium constant (expected for an endothermic process), but it is due to faster kinetics.

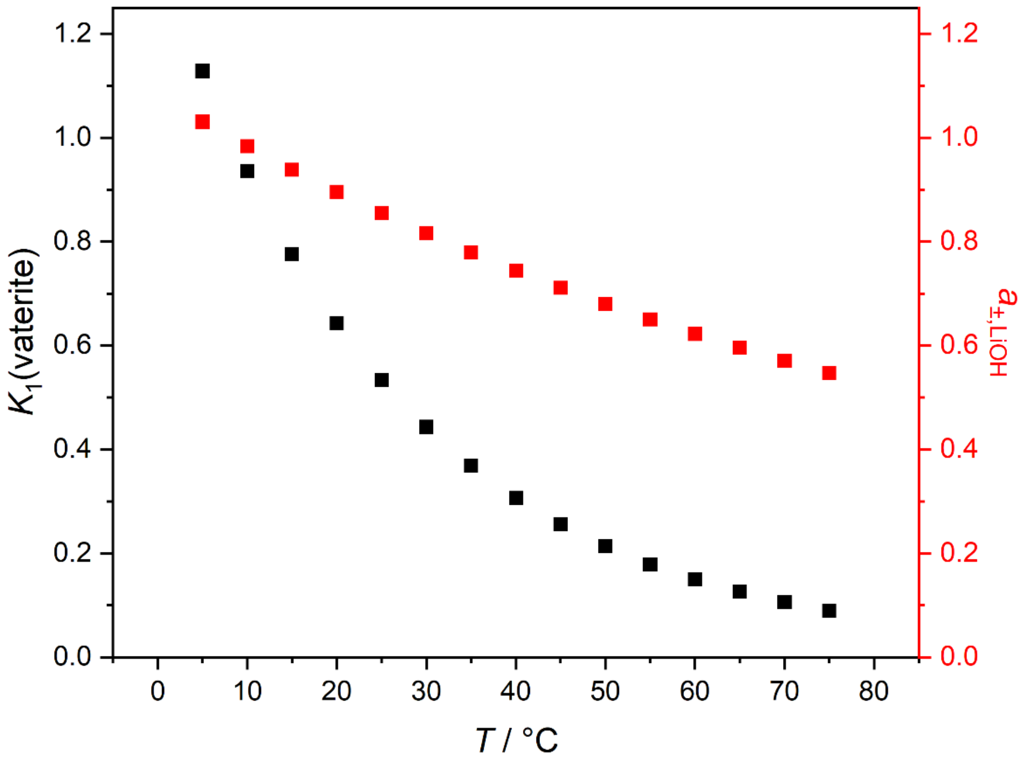

Figure S7 left axis: Equilibrium constant of the forward reaction assuming vaterite to be the equilibrium solid phase, K_1(vaterite), calculated from Eq. S10 based on Refs. [S6,S8, S13]. Right axis: 'mean' activity of LiOH solutios, obtained from K_1(vaterite) by Eq. S26.

11. Calculation of the mean activity coefficients

The mean activity coefficients $\gamma_{\pm,\text{LiOH}}^{\text{calc}}$, can be calculated from K_1(calcite) and K_1(vaterite) and corresponding $[\text{LiOH}]_{\text{eq}}$ concentrations according to Eq. S27. The thus obtained values can be found in Table 1 (main text). Experimental reference values $\gamma_{\pm,\text{LiOH}}^{\text{ref}}$ have been reported in Ref. [S14] as a function of molality. To calculate $\gamma_{\pm,\text{LiOH}}^{\text{ref}}$ at two specific $[\text{LiOH}]_{\text{eq}}$ concentrations, i.e. 1.6 and 2.3 mol L^{-1}, molalities in Ref. [S14] were converted to molarities using the equation in Ref. [S2]. Next, the $\gamma_{\pm,\text{LiOH}}^{\text{ref}}$ was fitted as a function of $[\text{LiOH}]_{\text{T}}$ by a biexponential function:

$$y = y_0 + a_1 e^{-b_1/[\text{LiOH}]_{\text{T}}} + a_2 e^{-b_2/[\text{LiOH}]_{\text{T}}} \quad \text{(S37)}$$

with fitted parameters y_0 = 0.4661(78), a_1 = 0.190(15), a_2 = 0.219(12), b_1 = –0.109(17), and b_2 = –1.07(17). Figure S8 clearly shows that all the data can be well described by this function, and $\gamma_{\pm,\text{LiOH}}^{\text{ref}}$ can readily be calculated by substituting $[\text{LiOH}]_{\text{eq}}$ = 1.6 or 2.3 mol L^{-1} for $[\text{LiOH}]_{\text{T}}$.

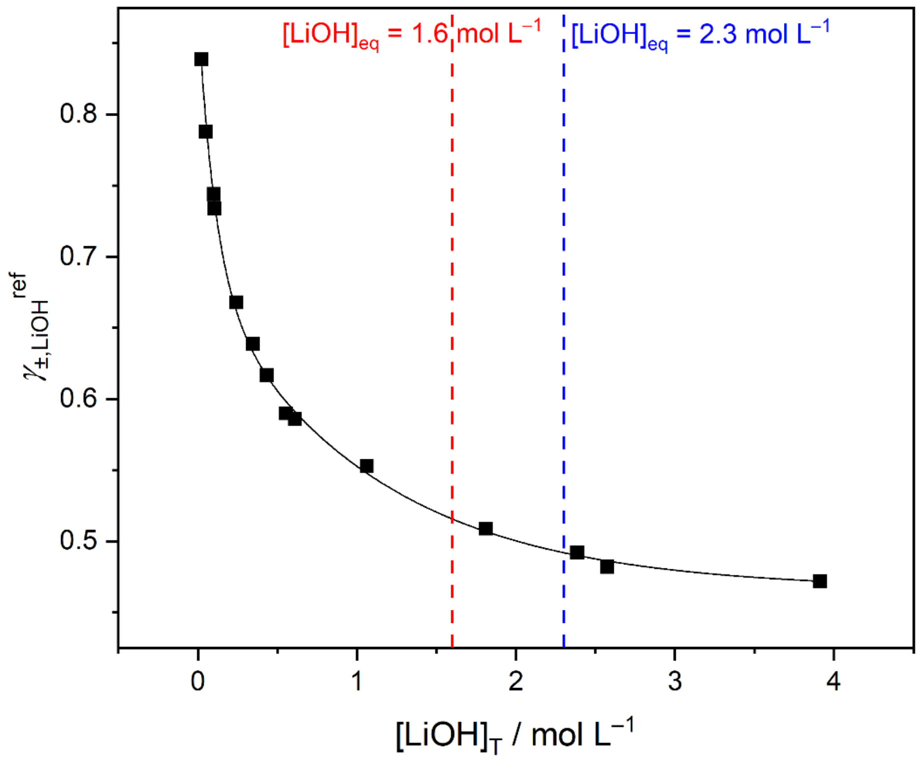

Figure S8 Reference values for the mean ionic activity coefficient of LiOH solutions, $\gamma_{\pm,LiOH}^{ref}$. Symbols represent experimental values from Ref. [S14], while solid line is the result of a biexponential fit (Eq. S35). Dashed lines correspond to the two equilibrium LiOH concentration with vaterite (red) or calcite (blue) being the equilibrium solid phase, in addition to Li_2CO_3 and $Ca(OH)_2$.

Note that the ~5% difference between $\gamma_{\pm,LiOH}^{calc}$ and $\gamma_{\pm,LiOH}^{ref}$ may be due to the assumption in Ref. [S16] that $\gamma_{\pm,LiOH} = 0.900$ for a 0.01 mol kg^{-1} LiOH solution. Further deviations may be related to the assumptions behind Eq. S26, and to the accuracy of K_1. For the latter, $K_{sp}(Li_2CO_3)$ is especially critical since literature data for this constant are sporadic. On the other hand, the difference is not affected by the degree of possible ion-pairing between Li^+ and OH^- ions [S3], as it was not considered in this work and in Ref. [S14].

References

[S1] Gates-Rector, S.; Blanton, T. The Powder Diffraction File: A Quality Materials Characterization Database. *Powder Diffr.* **2019**, *34*, 352–360

[S2] Gierszewski, P. J.; Finna, P. A.; Kirk, D. W. Properties of LiOH and $LiNO_3$ Solutions. *Fusion Eng. Design* **1990**, *13*, 59–71

[S3] De Oliveira, D. M.; Bredt, A. J.; Miller, T. C.; Corcelli, C. A.; Ben-Amotz, D. Spectroscopic and Structural Characterization of Water-Shared Ion-Pairs in Aqueous Sodium and Lithium Hydroxide. *J. Phys. Chem. B* **2001**, *125*, 1439–1446

[S4] Corti, H. R.; Crovetto, R.; Fernández Prini, R. Aqueous Solutions of Lithium Hydroxide at Various Temperatures: Conductivity and Activity Coefficients. *J. Solution. Chem.* **1979**, *8*, 897–908

[S5] Yi, W.-T.; Yan, C.-Y.; Ma, P.-H. Crystallization Kinetics of Li_2CO_3 from $LiHCO_3$ Solutions. *J. Cryst. Growth* **2010**, *312*, 2345–2350

[S6] Cheng, W.; Li, Z.; Cheng, F. Solubility of Li_2CO_3 in Na-K-Li-Cl Brines from 20 to 90°C. *J. Chem. Thermodyn.* **2013**, *67*, 74–82

[S7] Ekberg, C.; Brown. P. L. *Hydrolysis of Metal Ions*; Wiley-VCH: Weinheim, 2016; Vol. 1, p. 201.

[S8] Plummer, N. L.; Busenberg, E. The Solubilities of Calcite, Aragonite and Vaterite in CO_2–H_2O Solutions Between 0 and 90°C, and an Evaluation of the Aqueous model for the System $CaCO_3$-CO_2-H_2O. *Geochim. Cosmochim. Acta* **1982**, *46*, 1011–1040

[S9] Debye, P.; Hückel, E. Zur Theorie der Elektrolyte. I. Gefrierpunktserniedrigung und verwandte Erscheinungen. *Phys. Z.* **1923**, *24*, 185–206

[S10] Pannacci, N.; Lemaire, E.; Lobry, L. DC Conductivity of a Suspension of Insulating Particles with Internal Rotation. *Eur. Phys. J. E* **2009**, *28*, 411–417

[S11] Grágeda, M.; González, A.; Alavia, W.; Ushak, S. Development and Optimization of a Modified Process for Producing the Battery Grade LiOH: Optimization of Energy and Water Consumption. *Energy* **2015**, *89*, 667–677

[S12] Yuan, B.; Wang, J.; Cai, W.; Yang, Y.; Yi, M.; Xiang, L. Effects of Temperature on Conversion of Li_2CO_3 to LiOH in $Ca(OH)_2$ Suspension. *Particuology* **2017**, *34*, 97–102

[S13] Kutus, B.; Gácsi, A.; Pallagi, A.; Pálinkó, I.; Peintler, G.; Sipos, P. A Comprehensive Study on the Dominant Formation of the Dissolved $Ca(OH)_{2(aq)}$ in Strongly Alkaline Solutions Saturated with Ca(II). *RSC Adv.* **2016**, *6*, 45231–45240

[S14] Harned, H. S.; Swindells, P. E. The Activity Coefficient of Lithium Hydroxide in Water and in Aqueous Lithium Chloride Solutions, and the Dissociation of Water in Lithium Chloride Solutions. *J. Am. Chem. Soc.* **1926**, *48*, 126–135
